# Epigenetic differences between monozygotic twins discordant for amyotrophic lateral sclerosis (ALS) provide clues to disease pathogenesis

**DOI:** 10.1371/journal.pone.0182638

**Published:** 2017-08-10

**Authors:** Paul E. Young, Stephen Kum Jew, Michael E. Buckland, Roger Pamphlett, Catherine M. Suter

**Affiliations:** 1 Division of Molecular Structural and Computational Biology, Victor Chang Cardiac Research Institute, Darlinghurst, NSW, Australia; 2 Discipline of Pathology, Sydney Medical School, Brain and Mind Research Institute, The University of Sydney, Camperdown, NSW, Australia; 3 Department of Neuropathology, Royal Prince Alfred Hospital, Camperdown, NSW, Australia; 4 Faculty of Medicine, University of New South Wales, Kensington, NSW, Australia; Centre of Genomic & Post Genomics, ITALY

## Abstract

Amyotrophic lateral sclerosis (ALS) is a devastating late-onset neurodegenerative disorder in which only a small proportion of patients carry an identifiable causative genetic lesion. Despite high heritability estimates, a genetic etiology for most sporadic ALS remains elusive. Here we report the epigenetic profiling of five monozygotic twin pairs discordant for ALS, four with classic ALS and one with the progressive muscular atrophy ALS variant, in whom previous whole genome sequencing failed to uncover a genetic basis for their disease discordance. By studying cytosine methylation patterns in peripheral blood DNA we identified thousands of large between-twin differences at individual CpGs. While the specific sites of differences were mostly idiosyncratic to a twin pair, a proportion involving GABA signalling were common to all ALS individuals. For both idiosyncratic and common sites the differences occurred within genes and pathways related to neurobiological functions or dysfunctions, some of particular relevance to ALS such as glutamate metabolism and the Golgi apparatus. All four classic ALS patients were epigenetically older than their unaffected co-twins, suggesting accelerated aging in multiple tissues in this disease. In conclusion, widespread changes in methylation patterns were found in ALS-affected co-twins, consistent with an epigenetic contribution to disease. These DNA methylation findings could be used to develop blood-based ALS biomarkers, gain insights into disease pathogenesis, and provide a reference for future large-scale ALS epigenetic studies.

## Introduction

Amyotrophic lateral sclerosis (ALS), also known as motor neuron disease, is a lethal adult-onset disease that causes progressive muscle weakness, with death usually 2 to 5 years after initial diagnosis [1]. About 10% of ALS is familial and attributable to germline mutations in a number of genes, but in the majority of patients (~90%) no other family member is affected, and the causes of most of this so-called sporadic form of ALS remains unknown. Genetic, epigenetic and environmental factors have all been suggested to play a role in ALS, with combinations of these proposed to contribute to a multi-staged etiology [2].

Although rare single or multiple genetic variants may underlie some cases of ALS [3, 4], much of the heritability of the disease remains to be found [5]. ALS hereditability estimates from twin studies are 38–78% [6] and in family studies are 40–45% [7], but in a meta-analysis of three genome-wide association studies of common SNPs the reported ALS hereditability was 21% [8], suggesting much of the hereditability of the disease remains hidden [9]. Attention has therefore turned to the possibility that epigenetic factors could contribute to ALS and its associated condition, frontotemporal dementia [10]. The fact that epigenetic changes may be therapeutically modified has driven much of the research in this area [11]. A limited number of unvalidated epigenetic studies of ALS have been undertaken, involving single genes such as *SOD1* and *VEGF* [12], small groups of genes such as those in the metallothionein family involved in detoxifying heavy metals [13], and genome-wide methylation analysis using microarrays [14]. However, the role of epigenetic variants in ALS remains unclear [15].

Assessing the epigenetic basis of any disease in outbred populations such as humans is difficult because benign genetic variation is a major confounder [16]. Furthermore, distinguishing germline epigenetic abnormalities from somatic changes secondary to either pre- or post-natal environmental influences is a challenge [17]. This is particularly relevant to standard case-control studies because a vast number of environmental influences come into play within a normal human lifetime. One way of addressing variability between subjects is to study disease-discordant monozygotic twins, who share at least the same genome, are exposed to a parallel intrauterine environment, and often have similar lifestyles. This is an appealing approach for ALS since ALS twin registry studies show the disease is discordant in over 90% of monozygotic twins [6, 18, 19], which implies that susceptibility to the disease has a major epigenetic or environmental component. Epigenetic differences are known to exist between monozygotic twins [20], and such co-twin differences have been linked to disorders as diverse as psoriasis [21], neurofibromatosis [22], and frontometaphyseal dysplasia [23].

In this study we explored the nature and extent of epigenetic changes in peripheral blood DNA from five sets of ALS-discordant monozygotic twins, in whom extensive demographic and environmental exposure data were available, and in whom no pathologic co-twin genetic differences had been found [24, 25]. We compared genomic DNA methylation patterns between these twins in both case-control and co-twin analyses and found that four of the five ALS-affected twins were epigenetically older than their co-twins, suggesting an acceleration of cell aging in this disease. We also found a large number of differentially methylated sites between twins, most of which occurred at isolated CpGs and cluster in common genes and pathways relating to neurobiological functions.

## Results

### Monozygotic twins discordant for ALS show no evidence of germline epimutation at known ALS genes

Ten individuals were included in this study: five individuals with a diagnosis of sporadic ALS, and their respective unaffected monozygotic twin siblings (**Table 1**). All of the twin pairs led remarkably similar lives, with four twin pairs having the same occupations. **Table 1**shows all the differing characteristics between twin pairs, taken from self-filled demographic and clinical questionnaires (see **S1 File** to view the questionnaire). To preserve subject confidentially we are not able to publish all these responses here, but researchers who wish to view these data can contact Dr R Pamphlett to obtain de-identified results.

**Table 1:** Differing characteristics of ALS and nonALS monozygotic twins.

Twins (ALS/nonALS)	Gender	Age	Diagnosis of affected twin	ALS discordance (years)	ALS twin	NonALS twin
Pair 1 (A/B)	Male	52	PMA^§^	10	Non-smoker	Ex-smoker
Pair 2 (C/D)	Male	60	ALS	9	Non-smoker, depression	Non-smoker, boat-building chemicals
Pair 3 (E/F)	Female	53	ALS	8	Ex-smoker, asthma	Ex-smoker, GORD, statin
Pair 4 (G/H)	Female	62	ALS^§^	8	Ex-smoker, textile chemicals	Current smoker, nephritis, COPD, breast cancer, NHL
Pair 5 (I/J)*	Female	69	ALS	7	Non-smoker, stroke, hepatitis A, alcohol	Non-smoker, breast cancer

Age: age at blood collection, ALS: classic ALS, COPD: chronic obstructive pulmonary disease; GORD: gastro-esophageal reflux disease, NHL: non-Hodgkin’s lymphoma (diagnosed after blood collection), PMA: progressive muscular atrophy variant of ALS

*: also have a nonALS dizygotic triplet sibling

^§^ diagnosis confirmed on *post mortem* neuropathological examination

The average difference in time between ALS onset in the affected twin and the current age of the unaffected twin was 8.4 years (range 7–10 years), implying a non-genetic etiology of ALS in the affected twin. Consistent with this, none of the twins harboured an expanded repeat length at the *C9orf72* locus [25]. Furthermore, previous whole genome sequencing failed to detect any other significant genetic variation between these co-twins, with no pathogenic point mutation, insertion/deletion, or structural alteration identified in the affected twins compared with their unaffected co-twin [24]. We therefore considered the possibility that the underlying predisposing defect in the affected twins may be epigenetic in nature: epigenetic differences are not uncommon between monozygotic twins, and available evidence suggests that many such differences may be present from birth [20]. We obtained representative cytosine methylation profiles on peripheral blood DNA for each individual using both Illumina 450K Infinium methylation arrays [26] and reduced representation bisulfite sequencing (RRBS) [27]. The 450K array assesses methylation at a pre-determined set of ~450,000 single CpG sites concentrated around gene promoters and gene bodies. RRBS assesses ~1% of the genome and ~1 million or more CpGs; it is complementary to the 450K array since it also captures many CpGs outside of CpG islands and allows allelic resolution of methylation patterns. In our RRBS libraries we obtained >10x coverage on an average of 2.2 million CpGs and ≥20x coverage on an average of 1.4 million CpGs for each sample. Statistics on each of the twin RRBS libraries can be found in **S1 Table**.

**Fig 1:**
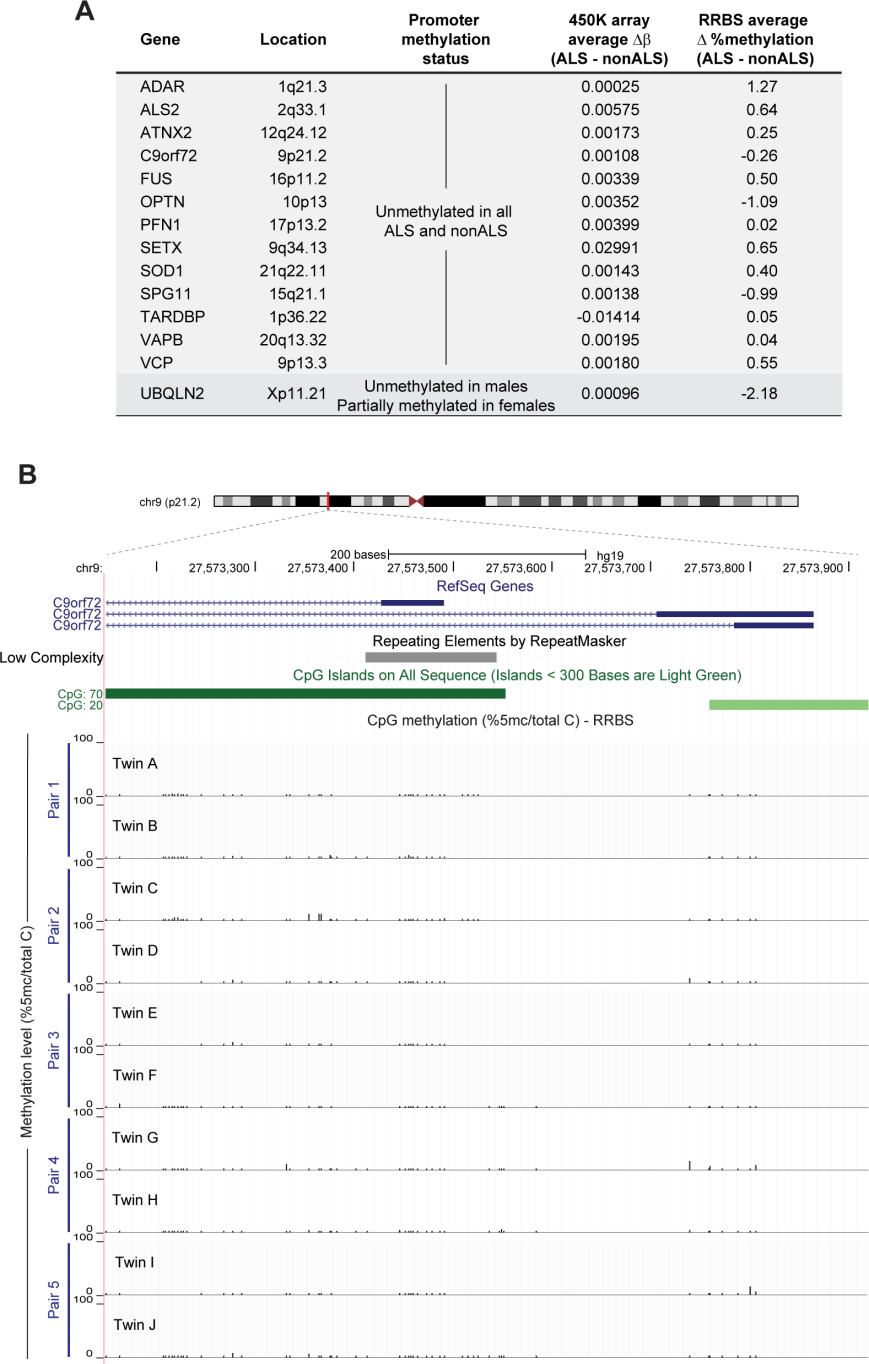
DNA methylation patterns at known ALS gene promoters do not differ between monozygotic twins discordant for ALS. **(A)** Known ALS disease genes captured by both Illumina Infinium 450K array and RRBS at 10x coverage; Δb represents the average difference in methylation levels between affected and unaffected twins. **(B)** Genome browser snapshot showing one representative example (the CpG island of C9orf72) of methylation patterns obtained by RRBS. The region harbouring the hexanucleotide repeat is shown by the grey bar under the RepeatMasker track. None of the twins harbour an expanded repeat, nor do they harbour significant methylation at any CpG across the C9orf72 promoter.

We first sought evidence for aberrant methylation in the affected twins at promoters of genes already implicated in ALS:*ALS2*, *ADAR*, *ATXN2*, *C9orf72*, *FUS*, *OPTN*, *PFN1*, *SETX*, *SOD1*, *SPG11*, *TARDBP*, *VAPB*, *VCP* and *UBQLN2* [28]. We found no evidence of differential methylation at probes within any known ALS gene promoter between affected and unaffected twins in the 450K array data. Further, at 10x coverage our RRBS libraries captured allelic information on the promoters of the same genes in all twin pairs, but none of the affected twins exhibited aberrant methylation at any of these loci (**Fig 1A**). Patterns of methylation at each known ALS disease locus were almost identical among all individuals, with all autosomal promoters showing little to no methylation, as shown, for example, in *C9orf72* (**Fig 1B)**. Methylation levels for all candidate gene promoters, and the precise regions captured by RRBS, are presented in **S2 File**. Taken together, these findings demonstrate that the discordance for ALS in these monozygotic twin pairs is not due to a germline genetic or epigenetic defect in any of the genes commonly associated with ALS.

### Case-control analysis of methylation implicates GABA receptor signalling as a commonly perturbed epigenetic network in ALS

**Fig 2:**
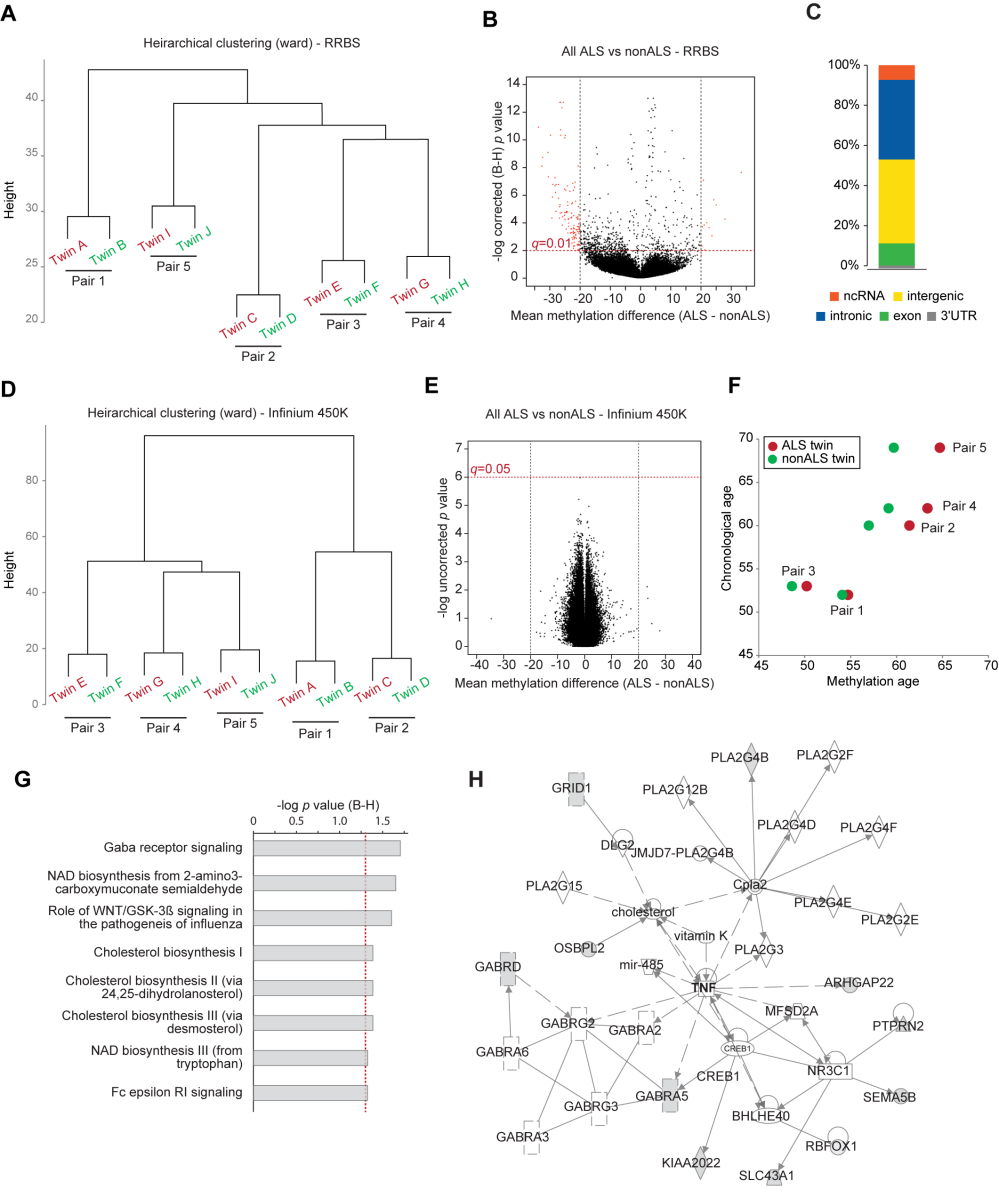
RRBS reveals commonly differentially methylated CpGs in ALS cases that cluster in GABA receptor signaling. **(A)** Dendrogram showing unsupervised hierarchical clustering of RRBS data at 10x coverage; ALS affected twin in red, nonALS co-twin in green. **(B)** Volcano plot showing mean methylation difference between ALS cases and controls (x-axis) versus -log corrected *p* values (y-axis) for CpG sites present in all RRBS libraries. Sites called as differentially methylated (at 20x coverage, 20% difference, q<0.01) are in red. **(C)** Genomic annotation of sites called as differentially methylated in (B). ncRNA: non-coding RNA. **(D)** Dendrogram showing unsupervised hierarchical clustering of Infinium 450K data. **(E)** Volcano plot showing mean methylation difference between ALS cases and controls (x-axis) versus -log uncorrected *p* values (y-axis) for all CpG sites present on the 450K array. **(F)** Plot showing chronological age versus methylation age for each twin pair; ALS affected twin in red, nonALS co-twin in green. **(G)** Top canonical pathways represented by the genes harbouring differentially methylated cytosines between ALS cases and controls. **(H)** Ingenuity Pathway Analysis network related to GABR signaling; genes harbouring differentially methylated cytosines are shaded grey.

We next took an unbiased approach to determine whether epigenetic differences underlie the twin discordance for ALS. Unsupervised hierarchical clustering of RRBS data at 10x did not separate cases and controls, but instead identified five distinct clusters representing the five twin pairs (**Fig 2A**). This is not surprising given the known influence of genotype on inherited methylation patterns [29]. We then used the statistical package methylKit [30] to ask whether any differentially methylated CpG sites (DMCs) were common to all ALS patients versus all unaffected controls. At a significance threshold of *q*<0.01, this identified 135 CpG sites with ≥20% average difference in methylation between the two groups (**Fig 2B; S2 Table**). About one half of these DMCs were in unannotated, intergenic regions of the genome, with the remainder predominantly within intronic regions (**Fig 2C**). Unsupervised clustering of the 450K data led to a similar clustering by twin pair, not disease status (**Fig 2D**). Analysis of the array data using minfi [31] failed to identify any significant common DMCs. CpGs with nominal significance, or approaching significance after correction for multiple testing, exhibited only minute differences in methylation between cases and controls (**Fig 2E**). Interestingly, however, application of the Horvath algorithm of epigenetic age to the 450K data [32] predicted that, in all twin pairs except twin pair 1 (with a PMA phenotype), the ALS-affected twin had a substantially older epigenetic age than their unaffected co-twin (**Fig 2F**).

None of the common DMCs identified by the RRBS case-control analysis exhibited changes consistent with a germline event, i.e., affecting most or all cells. On average the differences between cases and controls were less than 25%, and while mosaicism for a germline change cannot be ruled out in this study of a single tissue, it is more likely that these modest changes indicate common somatic changes in ALS-affected individuals that are consequent to their disease. Ingenuity Pathway Analysis [33] of the genes harbouring DMCs (*n* = 74) revealed enrichment for several pathways, the most significantly enriched being ‘GABA receptor signalling’ (**Fig 2G**). Ingenuity Pathway Analysis also identified four separate gene networks involving the affected genes (**S1 Fig**). The network containing genes involved in GABA signalling, shown in **Fig 2H**, centred around TNF. The other three pathways (two headed by cancer, and one by lipid metabolism) (**S1 Fig**) have no obvious pathogenetic link to ALS, but since so little is known about the cause of ALS these networks warrant further investigation.

### Outlier analysis of RRBS data reveals characteristic epigenetic differences between ALS and nonALS twins

While the RRBS case-control analyses revealed interesting changes common to all twins, the necessary grouping of individuals for analysis means large changes of potential biological significance in only one or two ALS-affected individuals would be lost to statistical analysis. The ‘power of the twin’ would also be lost; this is particularly relevant in epigenetic studies, where underlying DNA sequence can influence or even determine epigenetic state [34]. Given the clinical and genetic heterogeneity of ALS, the pathogenesis of motor neuron loss may be distinct in each affected twin. RRBS methylation patterns were therefore compared between each affected and unaffected individual in co-twin analyses.

**Fig 3:**
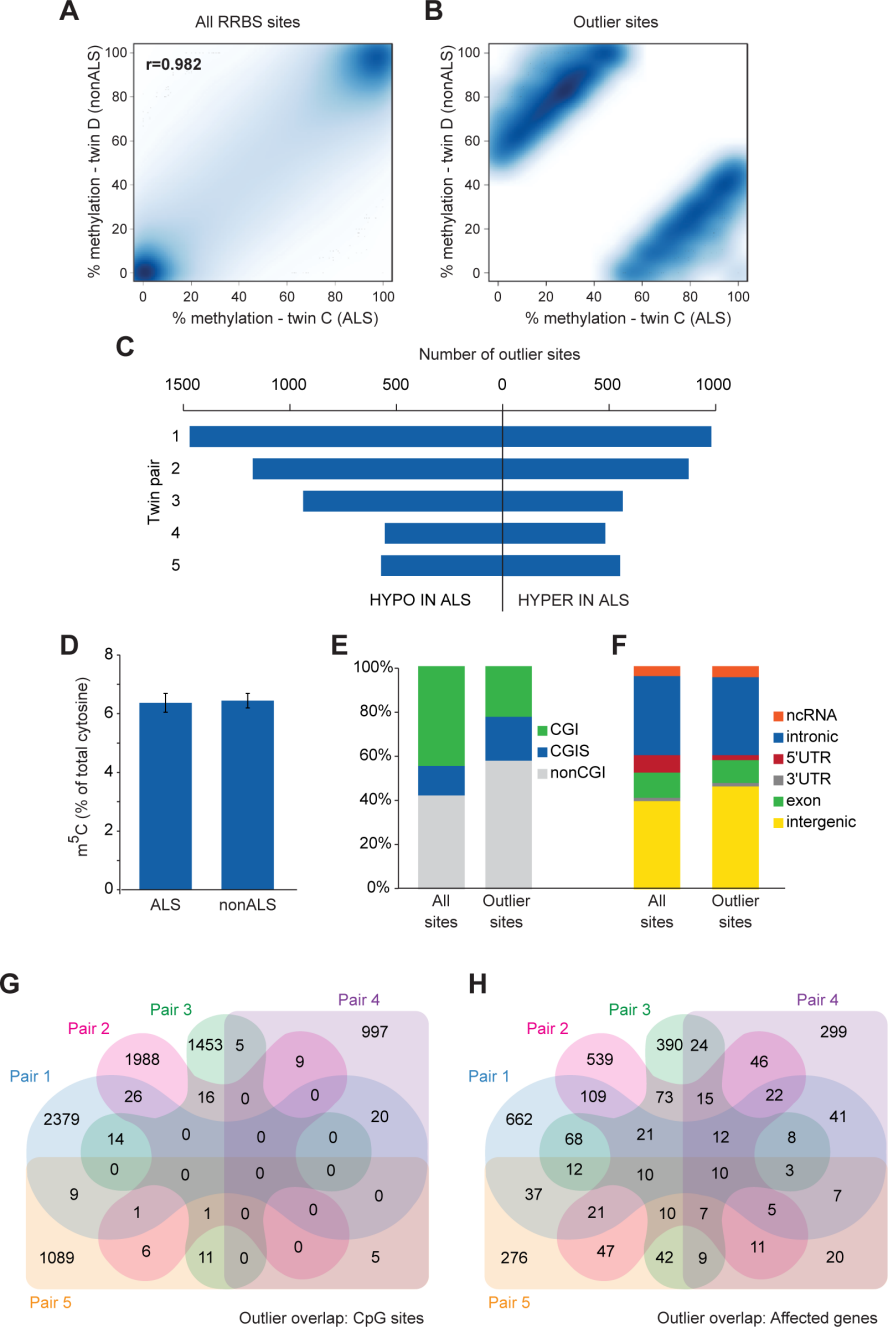
Thousands of CpG sites show great discordance in methylation between ALS discordant co-twins. **(A)** Smoothed correlation heatmap of all RRBS sites at 20x coverage in a representative twin pair (pair 2). **(B)** Smoothed correlation heatmap as in (A) showing only outlier sites ≥5 residuals from the linear model. **(C)** Bar graph showing the number of outliers defined by residuals in each twin pair. **(D)** Bar graph showing the total 5-methylcytosine content of peripheral blood DNA in ALS and nonALS individuals as measured by LC-MS/MS; error bars represent SEM. **(E,F)** Annotations for all RRBS sites and outlier sites for CpG islands (CGI) **(E)** and genomic location **(F)**. **(G,H)** Venn diagrams showing overlaps among twin pairs for individual CpG outliers **(G)** and genes harbouring outliers **(H)**.

We began by performing a Pearson’s correlation of methylation levels between co-twins. Co-twin CpG methylation was highly correlated overall (r = 0.978, range 0.972–0.982), and showed a generally bimodal distribution with most sites being either heavily methylated or largely unmethylated (**Fig 3A**). CpG sites present at >20x coverage in both twins within a pair were considered for further analysis. Those CpGs ≥5 residuals from the expected value from a linear model of all sites were called as methylation ‘outliers’ (**Fig 3B**). The minimum magnitude of difference in methylation at outliers between co-twins at this stringent cut-off was ~40%. Using this approach we identified more than 1,000 methylation outliers in each twin pair (**Fig 3C**; **S3 Table**). Although there was a preponderance for methylation outliers to be hypomethylated in the ALS twins relative to the non-ALS twins, whole genome levels of 5-methylcytosine, measured by liquid chromatography-tandem mass spectrometry (LC-MS/MS), did not differ between affected and unaffected individuals (**Fig 3D**), as has been previously suggested for ALS [35].

Genomic annotation of the outliers showed that, relative to all sites captured by RRBS, outlier sites were less likely to be in a CpG island (**Fig 3E**). Like the common DMCs identified by methylKit, outlier sites were predominantly in intronic and intergenic regions (**Fig 3F**). The majority of outlier CpGs were idiosyncratic to a twin pair, with little overlap among the twin pairs (**Fig 3G**). But when considering the genes harbouring the outlier CpG sites, the overlap among twins was greater, with ten genes (*ABR*, *NCOR2*, *SORCS2*, *HDAC4*, *SHANK2*, *RBFOX3*, *RXRA*, *MAD1L1*, *PTPRN2*, *GRIN1*) harbouring one or more methylation outliers in all five twin pairs (**Fig 3H**). Despite this overlap at the gene level, at least half of the affected genes were unique to a twin pair. **S3 File** provides a guide to searching for affected genes of interest in S2 and S3 Tables.

### ALS methylation outliers cluster in disease-relevant ontologies and pathways

We next took the genomic coordinates of the outlier CpGs and used the Genomic Regions Enrichment of Annotations Tool (GREAT) [36] to identify the ontologies of the sets of outliers for each twin pair. The molecular functions overrepresented by the outliers had one ontology in common across all twin pairs, ‘sequence specific DNA binding’ (**Table 2**). This is not disease-specific, but suggests that genes encoding transcription factors are susceptible to varying in epigenotype between identical genotypes. The significantly enriched biological functions revealed a large number of associated ontologies (**S4 Table**), many of which may be relevant to disease. With the exception of twin pair 2, outliers of all twin pairs exhibited enriched biological functions that cluster in neurobiological pathways, including dorsal spinal cord development and neuronal development and differentiation (**Table 3**). Cellular compartment ontologies of the outliers were significantly enriched in three of the five twin pairs, all of which share a ‘Golgi lumen’ compartment enrichment (**Table 4)**. Of note, Golgi fragmentation is a well-recognised early event in multiple *in vitro* and animal models of ALS [37].

**Table 2:** Molecular functions associated with ALS twin methylation outliers.

Molecular Function	Raw P-Value	FDR Q-Value	Fold Enrichment
**Twin Pair 1**			
sequence-specific DNA binding	4.14E-19	9.55E-17	2.16
**Twin Pair 2**			
sequence-specific DNA binding transcription factor activity	2.17E-23	6.66E-21	2.16
nucleic acid binding transcription factor activity	2.58E-23	7.32E-21	2.16
sequence-specific DNA binding	7.76E-22	2.05E-19	2.39
regulatory region DNA binding	1.51E-13	2.06E-11	2.45
**Twin Pair 3**			
sequence-specific DNA binding	7.01E-14	1.08E-11	2.25
tetrahydrobiopterin binding	5.79E-07	3.45E-05	21.05
**Twin Pair 4**			
DNA binding	3.09E-25	2.28E-22	2.18
sequence-specific DNA binding	6.68E-18	3.08E-15	2.81
sequence-specific DNA binding transcription factor activity	2.88E-15	1.18E-12	2.31
nucleic acid binding transcription factor activity	3.18E-15	1.17E-12	2.31
regulatory region DNA binding	5.65E-13	1.49E-10	3.10
transcription regulatory region DNA binding	2.65E-12	6.52E-10	3.05
transcription regulatory region sequence-specific DNA binding	7.76E-07	7.15E-05	3.09
**Twin Pair 5**			
transmembrane transporter activity	6.56E-11	3.02E-08	2.11
sequence-specific DNA binding	7.12E-11	2.92E-08	2.25
substrate-specific transmembrane transporter activity	8.30E-11	3.06E-08	2.17
ion transmembrane transporter activity	1.18E-10	3.96E-08	2.20
extracellular matrix structural constituent	3.83E-08	6.42E-06	4.57
cation channel activity	6.66E-07	7.23E-05	2.54

**Table 3:** Neurobiological^a^ processes associated with ALS twin methylation outliers.

Biological process	Raw P-Value	FDR Q-Value	Fold Enrichment
**Twin Pair 1**			
cell differentiation in spinal cord	4.46E-10	1.59E-08	4.00
spinal cord development	2.22E-09	7.30E-08	3.18
dorsal spinal cord development	5.14E-09	1.61E-07	6.25
spinal cord association neuron differentiation	5.10E-06	8.95E-05	5.79
**Twin Pair 3**			
generation of neurons	1.21E-23	2.21E-21	2.21
neurogenesis	3.95E-23	6.65E-21	2.16
neuron differentiation	2.33E-20	3.29E-18	2.28
neuron development	1.08E-14	9.83E-13	2.18
neuron projection morphogenesis	5.78E-12	3.55E-10	2.25
central nervous system development	9.64E-12	5.78E-10	2.02
**Twin Pair 4**			
central nervous system development	8.78E-14	1.01E-11	2.39
brain development	6.27E-12	5.85E-10	2.49
**Twin Pair 5**			
nervous system development	9.48E-20	3.96E-17	2.00
generation of neurons	1.63E-13	3.09E-11	2.01
regulation of nervous system development	3.74E-08	3.15E-06	2.19
regulation of neurogenesis	1.61E-07	1.19E-05	2.20
negative regulation of neuron differentiation	5.73E-06	2.74E-04	4.43

^**a**^ A full list of all enriched biological process can be found in S4 Table

**Table 4:** Cellular components associated with ALS twin methylation outliers.

Cellular Component	Raw P-Value	FDR Q-Value	Fold Enrichment
**Twin Pair 1**			
Golgi lumen	6.55E-16	2.37E-14	4.85
**Twin Pair 4**			
extracellular matrix	2.18E-12	7.06E-11	2.24
proteinaceous extracellular matrix	4.28E-11	1.32E-09	2.27
anchoring junction	3.77E-10	1.04E-08	2.62
adherens junction	1.83E-09	4.82E-08	2.61
Golgi lumen	8.03E-09	1.92E-07	3.82
cell-cell adherens junction	4.23E-05	0.000547	3.33
**Twin Pair 5**			
extracellular matrix part	1.74E-05	4.78E-04	2.53
Golgi lumen	5.22E-05	1.16E-03	3.62
voltage-gated potassium channel complex	7.20E-05	1.49E-03	3.51
cation channel complex	1.68E-04	2.95E-03	2.57
ion channel complex	1.78E-04	3.04E-03	2.16

**Fig 4:**
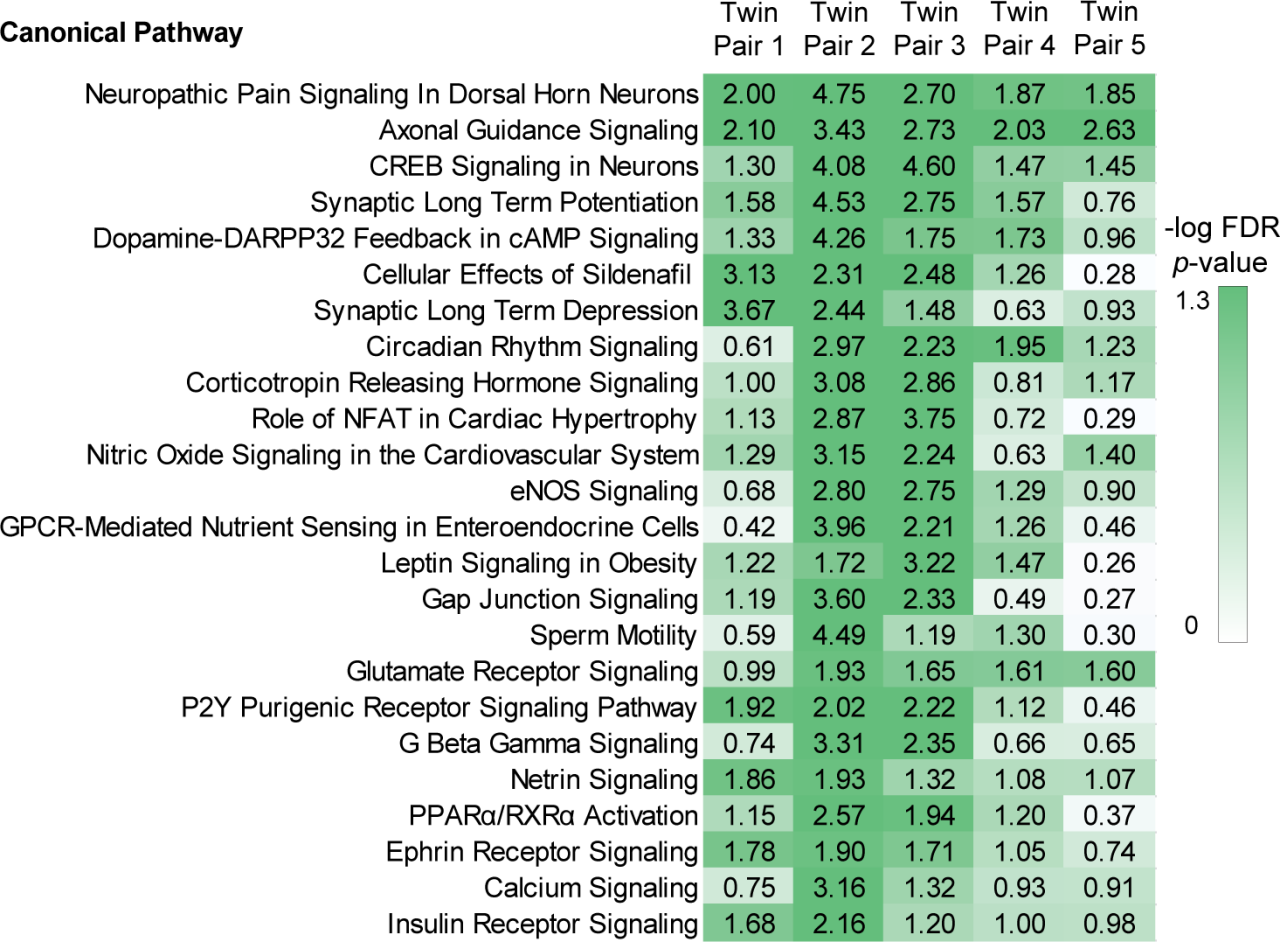
Many canonical pathways associated with ALS methylation outliers are common among twin pairs. Pseudo-heatmap showing top canonical pathways common to more than one twin pair; -log *p* >1.3 signifies significant enrichment.

Ingenuity Pathway Analysis of the genes harbouring methylation outliers produced a set of top canonical pathways for each twin pair (**S5 Table**). Cross-comparison of enriched pathways across all twin pairs revealed many significantly enriched pathways in common between two or more twin pairs (**Fig 4**). Most striking were the commonalities among neurobiological pathways, including pathways such as synaptic long-term potentiation. Taken together with the ontology analysis, this suggests that many methylation outliers represent an epigenetic signature of ALS in peripheral blood.

## Discussion

We have taken advantage of the genetic and early environmental similarity of identical twins discordant for ALS to gain insight into the nature and extent of epigenetic changes in this disease. Our findings demonstrate that ALS has epigenetic signatures in peripheral blood DNA that could potentially be exploited as biomarkers of disease. These findings are consistent with widespread disruptions to epigenetic patterns in ALS that either underlie disease etiology, or represent changes consequent to pathology.

Familial ALS is genetically heterogeneous, but clinically very similar to sporadic ALS, which prompted us to use our data to first examine methylation at genes known to be mutated in familial ALS. Germline epimutation, characterised by soma-wide aberrant silencing of a gene, can phenocopy a genetic mutation [38], and is usually associated with dense hypermethylation at the promoter of the affected gene. However none of the individuals exhibited any aberrant methylation at known ALS gene promoters in their peripheral blood. This finding does not necessarily preclude an inborn epigenetic defect as the basis for an affected twin’s predisposition to ALS, but it excludes this possibility at known ALS genes.

Unbiased case-control analyses are designed to detect commonalities between groups. It is of particular interest that our RRBS analyses revealed affected twin-concordant methylation changes at genes that cluster in GABA receptor signalling. Cortical hyperexcitability, one of the earliest identifiable changes in patients with ALS, is caused at least in part by degeneration of inhibitory cortical circuits and reduced cortical GABA levels [39, 40]. Given that ALS is a heterogeneous disease [41], however, these epigenetic changes common to all our ALS-affected twins could be to secondary to the many pathogenetic pathways operating in ALS, rather than being causally related to the disease. If so, these changes hold the potential to be exploited as blood-based biomarkers for an early diagnosis of ALS.

The finding of increased ‘epigenetic age’ in the white blood cells of all our four classic ALS patients supports the suggestion of a previous study of one ALS-discordant twin pair [42] that increased tissue aging may be a common feature in ALS. The changes to white blood cell methylation in a neurodegenerative disease such as ALS is consistent with recent findings that ALS is not a disorder of motor neurons alone, since other CNS cells such as astrocytes, oligodendrocytes, microglia, and interneurons, as well as skeletal muscle, are now implicated in its pathogenesis [43]. Furthermore, differences in non-neuromuscular organs are also found in ALS, such as changes in the skin which may explain the rarity of pressure ulcers in ALS patients despite prolonged immobility [44]. Our finding of accelerated methylation aging in ALS-discordant twins adds to the body of evidence that ALS is a truly systemic disorder. This widespread tissue involvement increases the likelihood that many cases of sporadic ALS are due either to *de novo* mutations [3], somatic mutations early in development [25], or exposure to environmental toxicants such as mercury that are taken up by multiple tissues [45]. Of interest, in our only twin pair who did not have substantially different methylation ages the affected twin had PMA, the form of ALS that is restricted to lower motor neurons. This raises the possibility that a somatic mutation later in development, or exposure to a toxicant with preferential CNS uptake, underlies this ALS variant.

When considering methylation differences between twins we found a considerable number of differences of large magnitude, and defined these as ‘methylation outliers’. Based on the magnitude of difference in methylation between co-twins at outliers and the stringent parameters we used to identify them, it is unlikely that these outliers reflect mere experimental noise. Indeed, for Twin Pair 2 we found that the methylation levels of outliers was highly correlated in two separate RRBS sequencing runs (**S2 Fig**). We do not expect, however, that all methylation outliers between co-twins will be representative of ALS discordance, since many differences may reflect or underlie other phenotypic discordances, or individual exposure to environmental factors [16]. For example, one of our individuals was a smoker at the time of sample collection and her co-twin was not; in this pair we were able to identify the expected difference in methylation levels at an intronic CpG in the AHRR gene, known to robustly associated with active smoking [46] (**S3 Fig**). This particular difference fell just under our outlier threshold of ≥5 residuals, but given that twin pairs carry thousands of outlier sites of greater magnitude than this, at least some of them will be expected to reflect the discordance for ALS, a supposition supported by the gene ontology and pathway analyses of outliers. Genome-wide analyses of outliers identified in healthy twins, performed in a similar manner [16], revealed between-twin differences that cluster largely in ontologies related to the tissue being examined; between-twin differentially methylated CpG sites in adipose tissue clustered in functions related to lipid metabolism while peripheral blood differentially methylated CpG sites clustered in haematological functions [16].

The thousands of outlier sites we identified in each twin pair showed only a modest overlap in genes affected, but all five twin pairs harboured outliers in ten common genes. Three of these genes have previously been implicated in ALS: *SORCS2*, *RXRA*, and *HDAC4*, which have prominent roles in inflammation and epigenetic regulation [47–49]. *GRIN1*, another of the ten common genes, encodes a subunit of the glutamate NMDA receptor, the major mediator of excitotoxicity; splicing of *GRIN1* requires the RNA binding protein TAF15, another molecule implicated in ALS [50]. The remaining genes, including *ABR*, *SHANK2*, *RBFOX3* and *PTPRN2* have no obvious link to ALS, but are notable for being highly expressed in the central nervous system. The genes which are affected in all our cases could be considered candidates in follow-up studies of larger ALS cohorts.

It is of interest that the most widespread epigenetic changes we found between ALS and nonALS twins were in two pathways considered by most researchers to underlie motor neuron death in ALS, i.e. glutamate-induced neuronal excitation and GABA-mediated neuronal inhibition. This topic has been extensively reviewed recently [51], with evidence from numerous studies showing how motor neuron survival in both the frontal motor cortex and the spinal cord depends on a delicate balance between synaptic stimulation (via glutamate) and inhibition (via GABA). Of note, the only therapeutic agent to slow the progress of ALS, riluzole, has a predominantly anti-glutamate action. A recent report of mercury uptake into human GABA-producing spinal interneurons [52], with the possibility that these damaged interneurons predispose the motor neurons to excitotoxic damage [53], emphases the importance of these pathways in ALS. Our findings of epigenetic differences in these pathways add further evidence to the proposal that excitotoxic motor neuron damage, which could be therapeutically modified, is an important mechanism in ALS.

The functional impacts of the ALS methylation outliers are difficult to tease out at present but they could, for example, affect local gene expression, expression *in trans*, or splicing. Whatever their function, the overlap in the gene networks and pathways affected was the most striking finding of this study. Neurobiological functions or pathways relevant to ALS were overrepresented in every twin pair, even with the modest lack of gene overlap, and more importantly, with the tissue that was examined (white blood cells, not CNS). We were not able to adjust for blood cell composition, but such differences, if present, would not be expected to result in enrichment for neurobiological-related ontologies. Perturbed neuro-related pathways in non-affected tissue might reflect different routes to the common endpoint of ALS in each affected twin. These could potentially be germline epigenetic changes that predispose to ALS, but we are unable to establish this because other tissues were not available for analysis. On the other hand, it is equally plausible, if not more likely, that the idiosyncratic CpG outliers in affected twins are representative of different environmental exposures, some of which contribute to ALS susceptibility. Assessing larger cohorts of ALS patients for the presence of the outliers identified in this study may yield greater insights into their role in this disease.

A noteworthy finding of this study is that the differences we identified with RRBS could not be detected with the 450K array, because the majority of ALS methylation outliers we found are not represented on the array. While the 450K array has been a popular method for epigenetic epidemiology due to its low cost and ease of analysis, our results show that the representative set of CpGs on the array are less than optimal in capturing the extent of epigenetic variation in ALS. RRBS captures only around 1% of the genome (although enriched for CpGs), but with the increasing affordability of high-throughput sequencing, whole genome bisulfite sequencing of large cohorts will soon be become feasible. Our results suggest that future whole genome bisulfite sequencing studies will be required to capture the full extent of epigenetic discordance among identical twins with discordant disease phenotypes.

## Materials and methods

### Ethics statement

Informed written consent was obtained from each individual for their DNA to be used in the study protocol ‘Looking for the Causes of MND’, approved by the Sydney South West Area Health Service Human Research Ethics Committee (no. X11-0383 & HREC/11/RPAH/601). Capacity to consent was judged in person by the investigator taking the blood sample on the basis of the individual: (1) being an adult over the age of 18 years, (2) being able to understand each section of the consent form, which was read out to them, with time for questions, and (3) having the intellectual ability to be capable of completing the demographic and environmental exposure questionnaire.

### Participants

Five individuals with a diagnosis of sporadic ALS and their ALS-unaffected monozygotic twin siblings were involved in this study. The diagnosis of ALS was made by a neurologist, with four having classic ALS (with upper and motor neuron signs) and one with the progressive muscular atrophy (PMA) variant (with lower motor neurons signs only). PMA is generally agreed to be a form of ALS [54], with TDP-43 inclusions also found in motor neurons in this variant [14], so for analysis purposes these two disorders were both considered to be ALS. Autopsy neuropathological confirmation of the diagnosis was available for one patient with classic ALS and one with the PMA variant. No twin had a family history of ALS. All affected and unaffected co-twins donated blood samples to the Australian Motor Neuron Disease DNA Bank and completed a detailed demographic and environmental exposure questionnaire. Epidemiological and clinical differences between the co-twins are shown in Table 1. Venous blood samples were taken from an antecubital vein at the same time in each twin pair. DNA was extracted from white blood cells using the QIAmp blood kit (Qiagen) and stored at -20°C until used.

### Total 5-methylcytosine (5mc) content

Total 5mc content of each DNA sample was analysed by liquid chromatography-mass spectrometry (LC-MS/MS). Approximately 1 μg of genomic DNA was used in hydrolysis using DNA Degradase Plus (Zymo). The reaction mixture was incubated at 37°C for two hours to ensure complete digestion prior to LC-MS/MS, as described previously [55].

### Reduced representation bisulfite sequencing (RRBS)

Indexed RRBS libraries were prepared from 1μg of *Msp*I-digested genomic DNA essentially as described [27], and sequenced in multiplex on the Illumina HiSeq 2000. Resulting fastq files were trimmed with cutadapt v1.3. Trimmed reads were aligned to the human reference genome (hg19) using Bismark v0.10.0 [56] paired with Bowtie v1 [57] with default parameters with methylation calling by Bismark-methylation-extractor. Output files were reformatted for direct input into methylKit using a custom script.

### RRBS case-control analysis

Differentially methylated CpG sites between all cases and controls were identified using the Bioconductor R package methylKit [30] with filter settings of ≥ 20X coverage, ≥ 20% methylation difference, and *q* value of 0.01.

### Outlier analysis

Linear models were established using R for each twin pair using methylation calls for CpG sites in common to co-twins with ≥20x coverage. Outlier CpG sites were defined as those ≥5 residuals from the predicted value from the linear model. Genomic coordinates for outlier sites for each twin pair were analysed with the gene ontology software GREAT [36]. Genes harbouring outliers were analysed further by Ingenuity Pathway Analysis (http://www.ingenuity.com/).

### Illumina Infinium 450K arrays

Infinium 450K arrays were performed on each sample by the Australian Genome Research Facility (http://www.agrf.org.au/). Resultant data were analysed using the Bioconductor package minfi [31] using SWAN normalisation. Only probes with a detection value of *p* value <0.01 were included in differential methylation analysis. Epigenetic age was calculated using the method of Horvath [32].

## Supporting information

S1 Fig Networks identified by IPA with genes harbouring differentially methylated cytosines in all ALS vs nonALS twins. (PDF)

S2 Fig Methylation levels at outlier sites are highly correlated in two independent RRBS runs. (PDF)

S3 Fig RRBS and 450K identify methylation differences known to associate with cigarette smoking. (PDF)

S1 File Patient questionnaire. (PDF)

S2 File Genome browser snapshots of regions captured by RRBS for each ALS-associated gene promoter listed in Fig 1A. (PDF)

S3 File Guide to searching the twin-related methylation status of a gene of interest (in S2 and S3 Tables). (PDF)

S1 Table CpG coverage in RRBS libraries. (PDF)

S2 Table RRBS sites detected as differentially methylated when comparing all ALS cases vs all matched unaffected twin siblings. (XLSX)

S3 Table Individual RRBS outlier sites for each twin pair. (XLSX)

S4 Table Gene ontology terms for RRBS outlier sites in each twin pair. (XLSX)

S5 Table Top canonical pathways of genes harbouring outlier sites. (XLSX)
