# Supplementary material for: Epigenetic differences between monozygotic twins discordant for amyotrophic lateral sclerosis (ALS) provide clues to disease pathogenesis
Source: PLoS One. 2017 Aug 10;12(8):e0182638. doi: 10.1371/journal.pone.0182638 (PMC5552194; doi:10.1371/journal.pone.0182638)
Supplement: S1 Fig — (PDF) [file pone.0182638.s001.pdf]

IPA network	Score	Focus Molecules	Top Diseases and Functions
1	54	23	Cancer, Gastrointestinal Disease, Organismal Injury and Abnormalities
2	31	15	Lipid Metabolism, Small Molecule Biochemistry, Cell-To-Cell Signaling and Interaction
3	26	13	Cancer, Organismal Injury and Abnormalities, Tissue Morphology
4	21	11	Connective Tissue Disorders, Developmental Disorder, Skeletal and Muscular Disorders

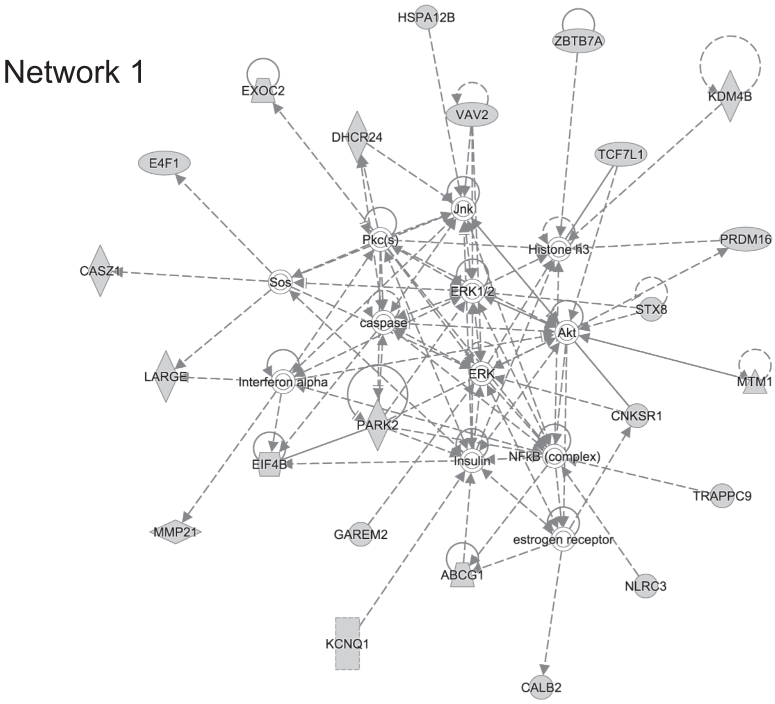

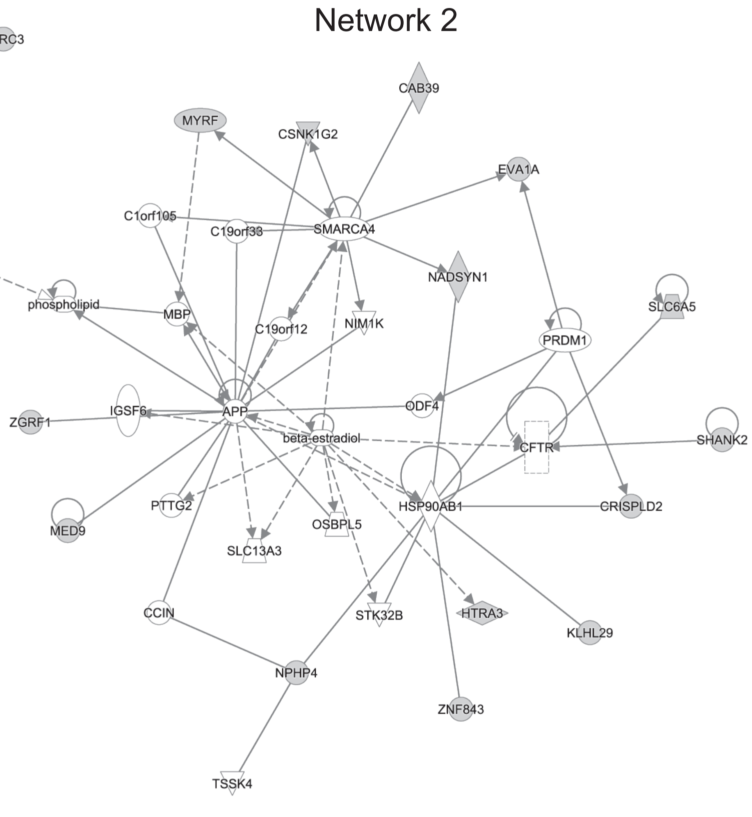

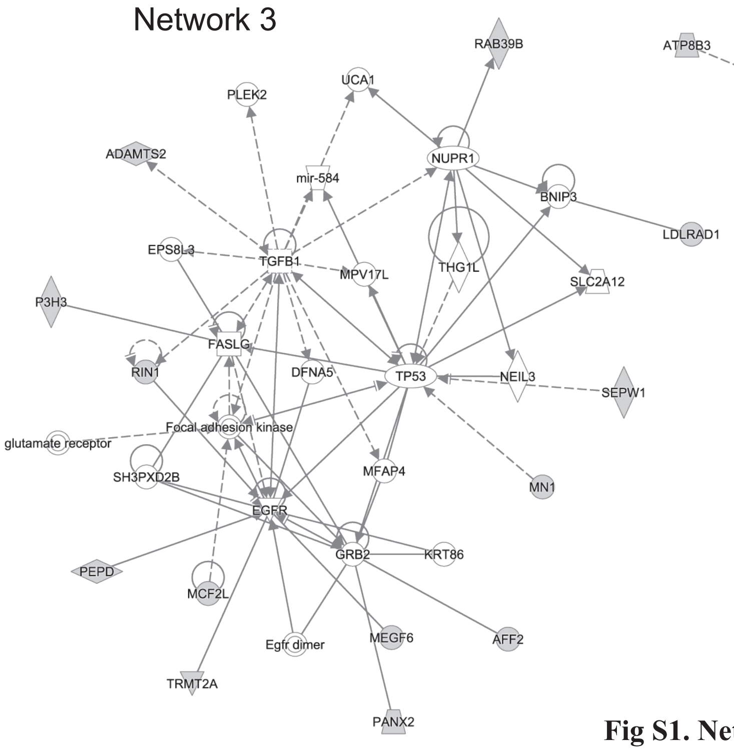

Fig S1. Networks identified by IPA with genes harbouring differentially methylated cytosines in all ALS vs nonALS twins. (Network 4 is shown in Fig 1).
