# Supplementary material for: Epigenetic differences between monozygotic twins discordant for amyotrophic lateral sclerosis (ALS) provide clues to disease pathogenesis
Source: PLoS One. 2017 Aug 10;12(8):e0182638. doi: 10.1371/journal.pone.0182638 (PMC5552194; doi:10.1371/journal.pone.0182638)
Supplement: S2 Fig — (PDF) [file pone.0182638.s002.pdf]

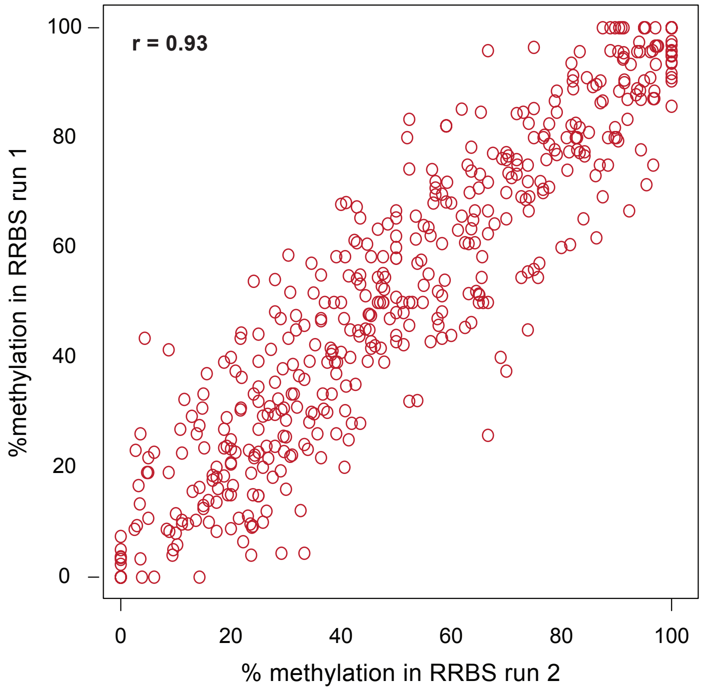

Fig S2. Methylation levels at outlier sites are highly correlated in two independent RRBS runs. Scatter plot showing methylation levels (5methylcytosine as a proportion of all cytosine) of outlier sites in twin pair 2 in two independent RRBS runs.
