# Supplementary material for: Epigenetic differences between monozygotic twins discordant for amyotrophic lateral sclerosis (ALS) provide clues to disease pathogenesis
Source: PLoS One. 2017 Aug 10;12(8):e0182638. doi: 10.1371/journal.pone.0182638 (PMC5552194; doi:10.1371/journal.pone.0182638)
Supplement: S3 Fig — (PDF) [file pone.0182638.s003.pdf]

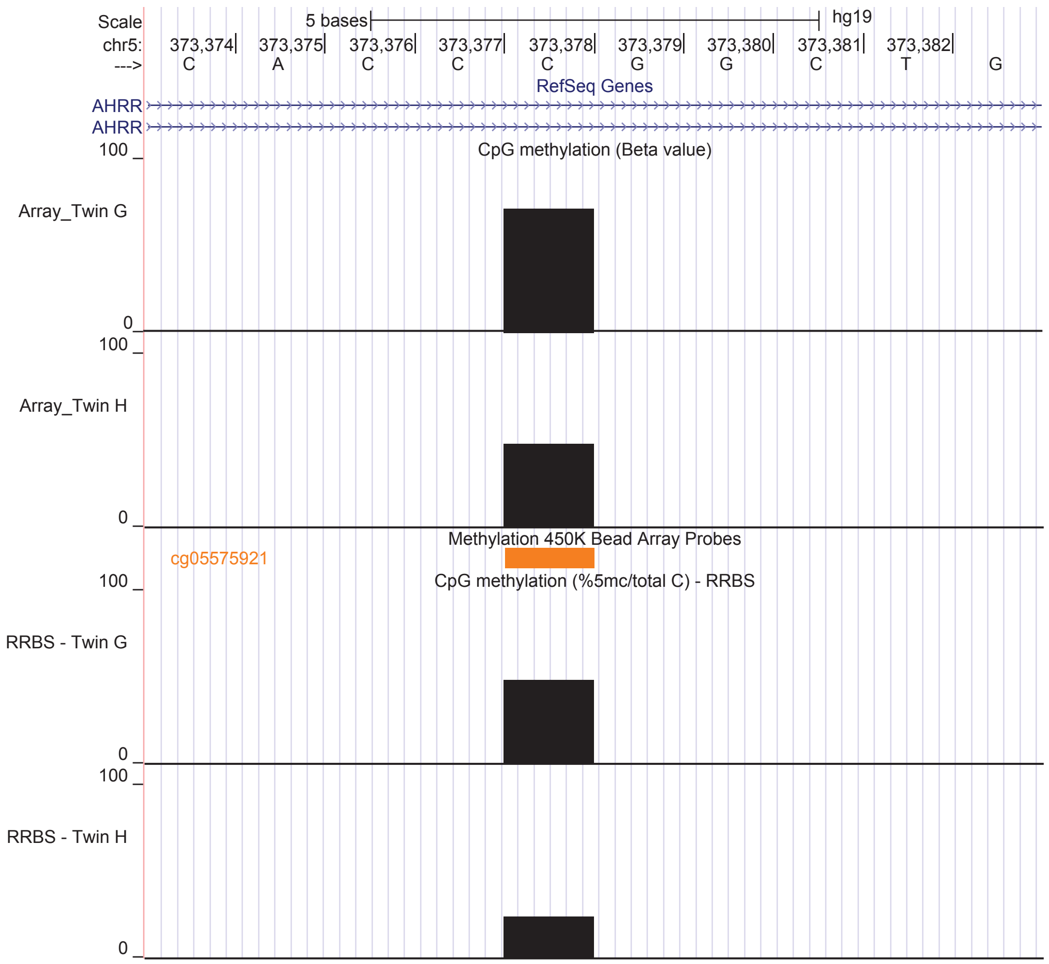

Fig S3. RRBS and 450K identify methylation differences known to associate with cigarette smoking. Genome browser snapshot showing methylation levels at a CpG site (cg05575921) in an intron of the gene encoding *aryl hydrocarbon receptor repressor* (*AHRR*). Methylation levels at this CpG have been reproducibly shown to be sensitive to smoking, with smokers presenting with lower levels of methylation than non-smokers. In Twin Pair 4, Twin H is a current smoker and Twin G is not. Methylation levels at cg05575921 are as expected in both RRBS and 450K array data with Twin H exhibiting lower methylation than Twin G.
