# Supplementary material for: Epigenetic differences between monozygotic twins discordant for amyotrophic lateral sclerosis (ALS) provide clues to disease pathogenesis
Source: PLoS One. 2017 Aug 10;12(8):e0182638. doi: 10.1371/journal.pone.0182638 (PMC5552194; doi:10.1371/journal.pone.0182638)
Supplement: S1 File — (PDF) [file pone.0182638.s004.pdf]

Strictly Confidential

The University Of Sydney

The Australian Motor Neuron Disease DNA Bank

Questionnaire for people who have motor neuron disease

Name ....

Address ....

....

Telephone/fax/email ....

....

Date of birth ....

Date of completing this form ....

Please circle the correct answers and complete to the best of your ability.

If you do not have enough space for any section, please use a separate piece of paper to complete.

The answers will be treated as strictly confidential.

Please forward completed form to A/Prof Roger Pamphlett, Department of Pathology D06, The University of Sydney, NSW 2006. Phone (02) 9351-3318, fax (02) 9351-3429, email roger.pamphlett@sydney.edu.au

Thank you for your co-operation

General

What is your gender? (Male/Female)

What is your ethnic background? (e.g. Irish, Indian) ....

Your father's ethnic background? ....

Your mother's ethnic background? ....

What race do you consider yourself to be? (e.g. white, black, Asian) ……………..

Father's date of birth?……………….. Mother's date of birth?…………………..

If your father is deceased, in which year did he die? ……………..

If your mother is deceased, in which year did she die? …………..

Did your father (Yes/No) or mother (Yes/No) ever smoke cigarettes?

Father's main profession? ....

Mother's main profession? ....

How many brothers and sisters (in total) do you have?………. Are you a twin? (Yes/No)

Where do you come in the order of births? (e.g. 3rd) ………..

Were you born in Australia? (Yes/No)

If "No" a) where were you born? ....

b) what year did you come to Australia?..

What is the highest level of education you have completed (circle one)?

a) primary school b) secondary school c) university degree

Lifetime residences

Have you ever lived in a non-city area for 12 or more months? (Yes/No)

Have you ever lived or worked on a farm for 12 or more months? (Yes/No)

List the places you have lived in for more than 2 years, from first (childhood) to last, giving the time you spent in each location. For each place, please indicate by "U" if urban (city or town) or "R" if rural (village or isolated house). Please attach an extra sheet if there are more places you have lived for more than 2 years.

Place lived (e.g. Canberra, farm near Dubbo NSW)	Years there	Urban or rural

Lifetime travel

List countries you have traveled to, giving the year of travel

Country	Year

Family illnesses

Has any member of your family (blood relatives) suffered from any of the following conditions?

Poliomyelitis.. (Yes/No)

Motor neuron disease .... (Yes/No)

Alzheimer's disease... (Yes/No)

Parkinson's disease .... (Yes/No)

Any other brain disease...................................... (Yes/No)

If "yes", specify which relative was affected and by what disease:

Relative	Disease

Lifetime employment

List the occupations you have had, from the first to the last, giving the number of years you spent in each occupation. Please note with an "M" if the occupation included manual work (i.e. if involving hard physical labour).

Occupation	Number of years	Manual?

Exposure to chemicals or toxins

1. ***Have you had exposure to solvents or chemicals at work?***..........................(Yes/No)

   If "yes", what chemicals or solvents did you work with?

   ....

   ....

2. ***Have you ever worked in the extraction of minerals, ores, or the manufacture of metals?*** .... (Yes/No)

3. ***As part of your occupation, have you ever worked with:***

   Lead ....(Yes/No)

   Mercury................(Yes/No)

   Cadmium..............(Yes/No)

   Copper.................(Yes/No)

   Any other mineral/metal? (specify) ....

4. ***Have you ever been exposed to herbicides or pesticides?*** ....(Yes/No)

   If "yes" to herbicides or pesticides, was the exposure:

   a) Occasional

   b) Regular (weekly exposure for more than six months)

   If you have been exposed to herbicides or pesticides, how were you exposed?

   a) Hobby gardening b) Farming c) Industrial or factory

   d) Other (specify)..

   Which herbicides or pesticides were you exposed to?

   ....

   ....

5. ***Have you used well-water, spring-water or bore-water as your main source of drinking water for more than six months?***…….(Yes/No)

Injuries

List the times you have had injuries (either bone fractures, blunt injury, operations with general anesthetic, or electric shocks) severe enough to require medical attention. Record the year, site and nature of the injury.

Year of injury	Part of body injured	Nature of injury

Physical exercise

Have you ever had a period of prolonged physical activity (e.g. athletics training) over a period of months?..(Yes/No)

What type of physical activity was it, and what year(s) was it?

....

....

....

Personal habits

Have you even been a cigarette smoker?................. (Yes/No)

In "yes", are you a:

a) Ex-smoker

What age did you start? ....

What age did you stop?..

How many cigarettes/day did you smoke? ....

b) Current smoker

What age did you start? ....

How many cigarettes/day do you smoke? ....

Alcohol consumption (please circle one response for each of the next three questions):

a) How often do you have a drink containing alcohol?

Never Monthly or less 2-4 times a month 2-3 times a week 4 or more times a week

b) How many drinks containing alcohol do you have on a typical day when you are drinking?

1 or 2 3 or 4 5 or 6 7 to 9 10 or more

c) How often do you have six or more drinks on one occasion?

Never Less than monthly Monthly Weekly Daily or almost daily

List any hobbies or pastimes that you have (e.g. sports, collections)

....

....

....

....

....

List any pets that you have kept in your house in the past (e.g., cats, dogs)

....

....

....

....

....

Past illnesses

Do you suffer (or have you ever suffered) from any of the following? If so, give the year it was diagnosed

Stroke ....

Heart attack ....

High blood pressure ....

Other major illnesses (with year of diagnosis)...

....

....

....

....

....

....

....

What medications have you used regularly (e.g. anti-ulcer medications)

Name of medication	Year started	Year finished	Still taking

			(tick)

Past medications used occasionally (e.g. aspirin)

………

………

………

………

………

………

………

………

………

………

………

………

Only people with Motor Neuron Disease need to complete this page of the questionnaire

What year did you get your first *symptom* of Motor Neuron Disease?............................

What was the first thing you noticed going wrong, and what part of your body did it affect?

....

....

....

What year were you *diagnosed* with Motor Neuron Disease?………………………..

What is the name and address of your general practitioner?

....

....

What is the name and address of your neurologist?

....

....

This is the end of the questionnaire. Thank you for your help with this research.
