# Supplementary material for: Epigenetic differences between monozygotic twins discordant for amyotrophic lateral sclerosis (ALS) provide clues to disease pathogenesis
Source: PLoS One. 2017 Aug 10;12(8):e0182638. doi: 10.1371/journal.pone.0182638 (PMC5552194; doi:10.1371/journal.pone.0182638)
Supplement: S2 File — (PDF) [file pone.0182638.s005.pdf]

Supplementary file 2. Genome browser snapshots of regions captured by RRBS for each ALS-associated gene promoter listed in Figure 1A. For each browser snapshot the CpG sites covered in each twin are indicated by the grey shaded vertical bars. CpG islands are shown in green. The snapshots are followed by a summary table of coverage and average methylation across each region shown in the browser snapshots.

ADAR - Chr 1

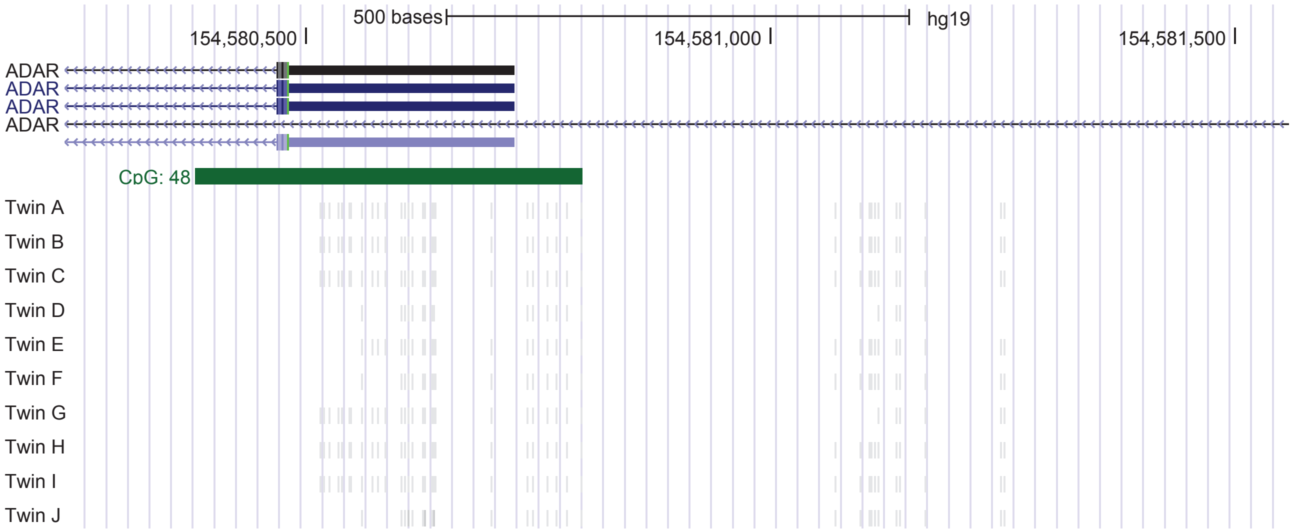

ALS2 - Chr 2

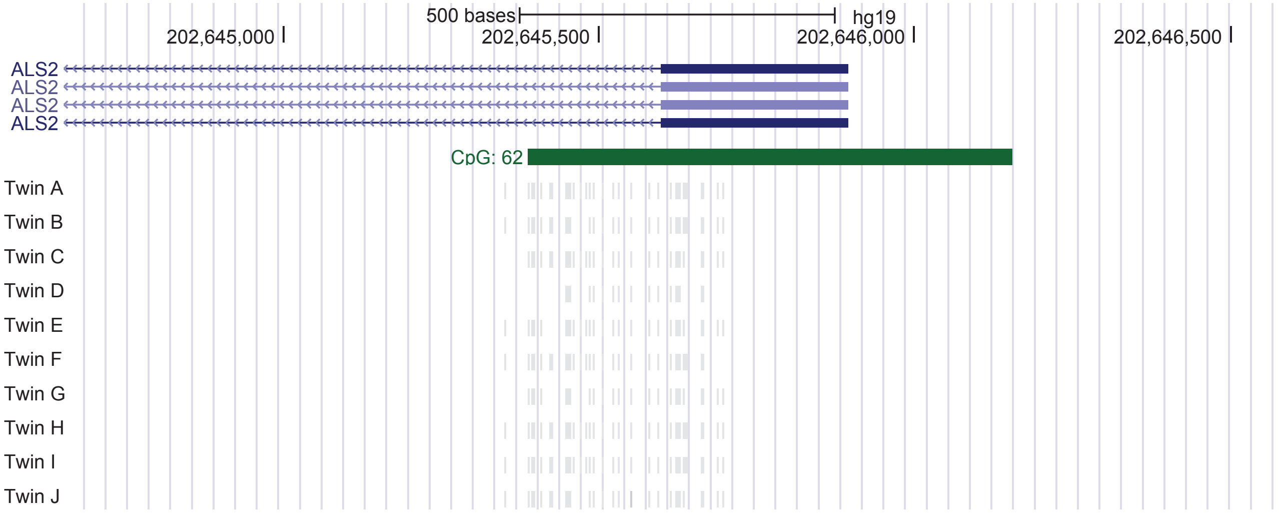

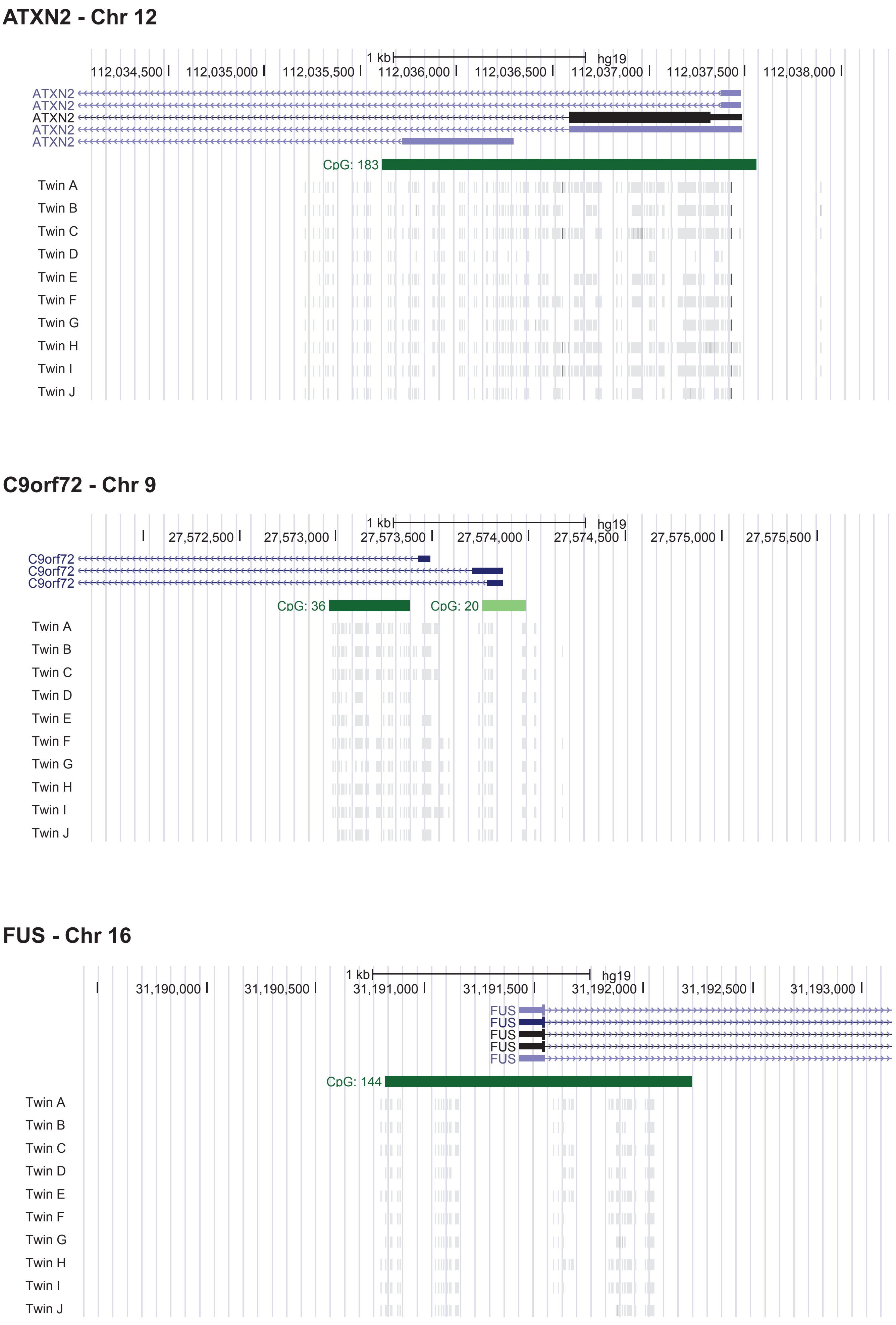

ATXN2 - Chr 12
1 kb
hg19
112,034,500
112,035,000
112,035,500
112,036,000
112,036,500
112,037,000
112,037,500
112,038,000
ATXN2
ATXN2
ATXN2
ATXN2
ATXN2
CpG: 183
Twin A
Twin B
Twin C
Twin D
Twin E
Twin F
Twin G
Twin H
Twin I
Twin J
C9orf72 - Chr 9
1 kb
hg19
27,572,500
27,573,000
27,573,500
27,574,000
27,574,500
27,575,000
27,575,500
C9orf72
C9orf72
C9orf72
CpG: 36
CpG: 20
Twin A
Twin B
Twin C
Twin D
Twin E
Twin F
Twin G
Twin H
Twin I
Twin J
FUS - Chr 16
1 kb
hg19
31,190,000
31,190,500
31,191,000
31,191,500
31,192,000
31,192,500
31,193,000
FUS
FUS
FUS
FUS
FUS
CpG: 144
Twin A
Twin B
Twin C
Twin D
Twin E
Twin F
Twin G
Twin H
Twin I
Twin J

OPTN - Chr 10

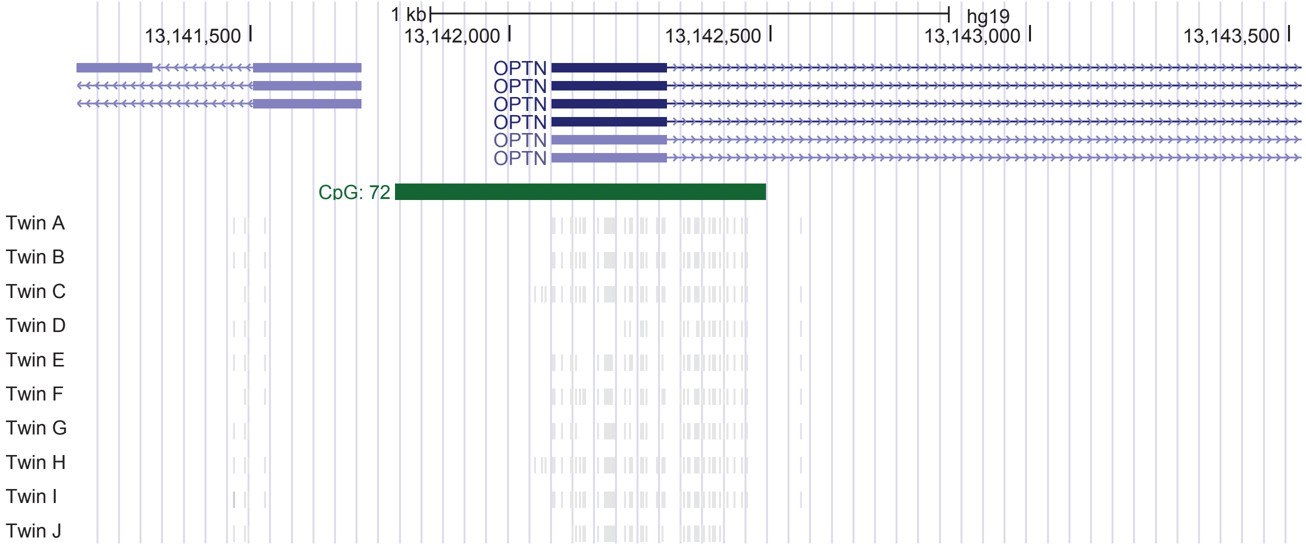

PFN2 - Chr 17

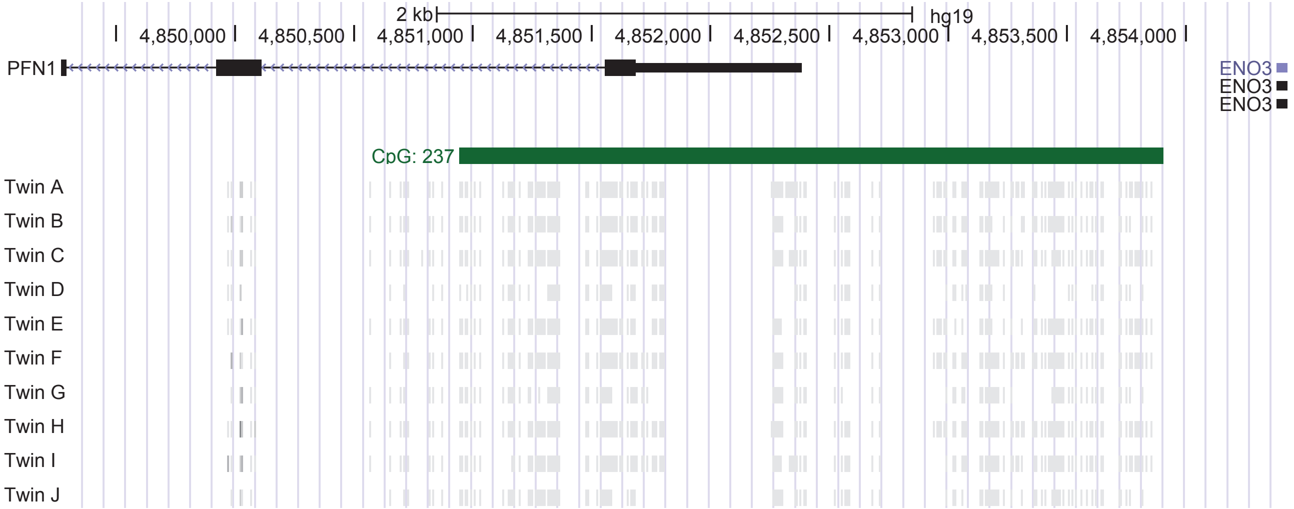

SETX - Chr 9

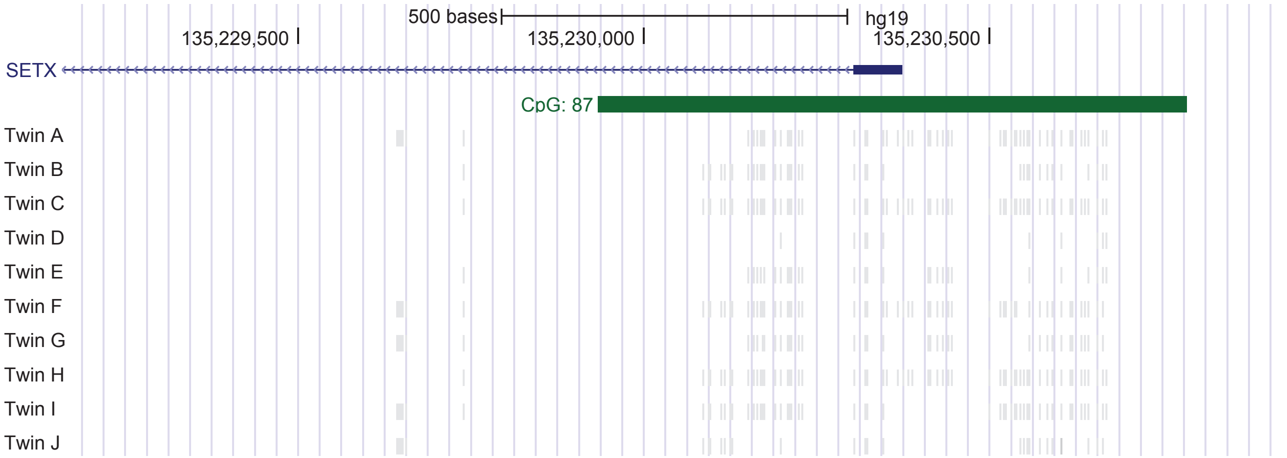

SOD1 - Chr 21

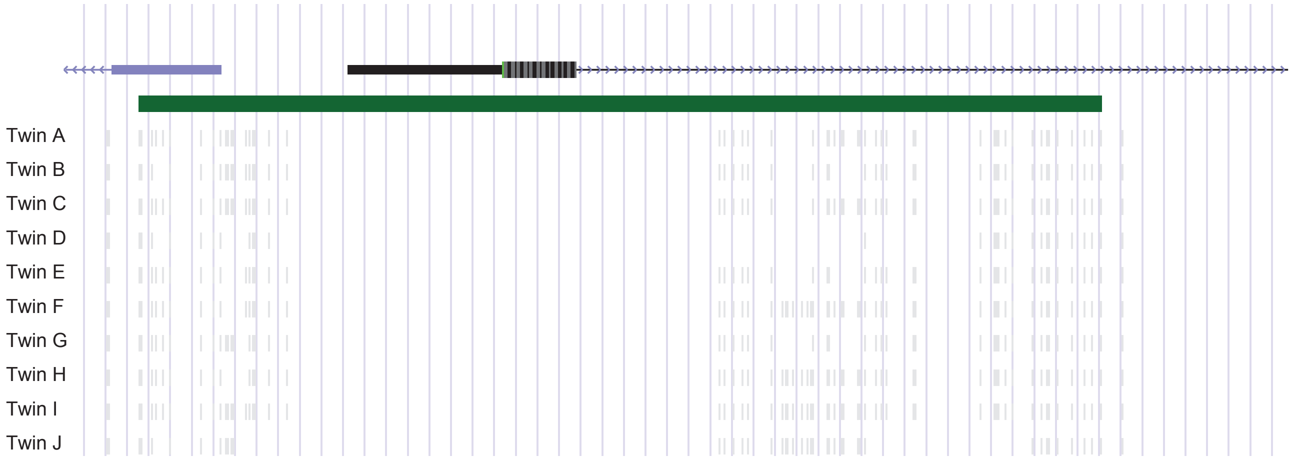

SPG11 - Chr 15

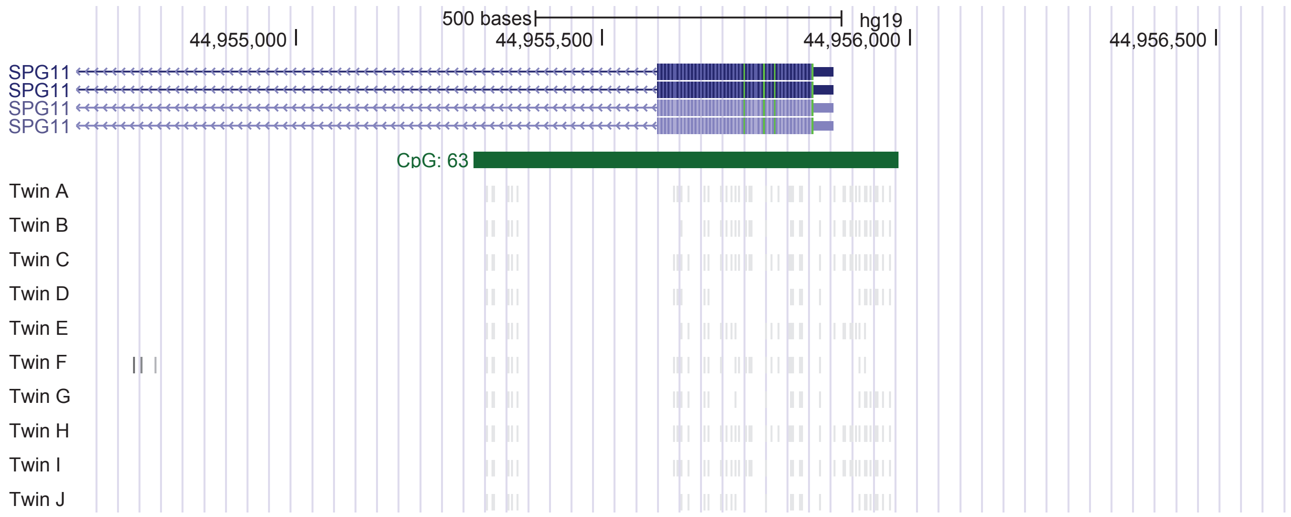

TARDBP - Chr 1

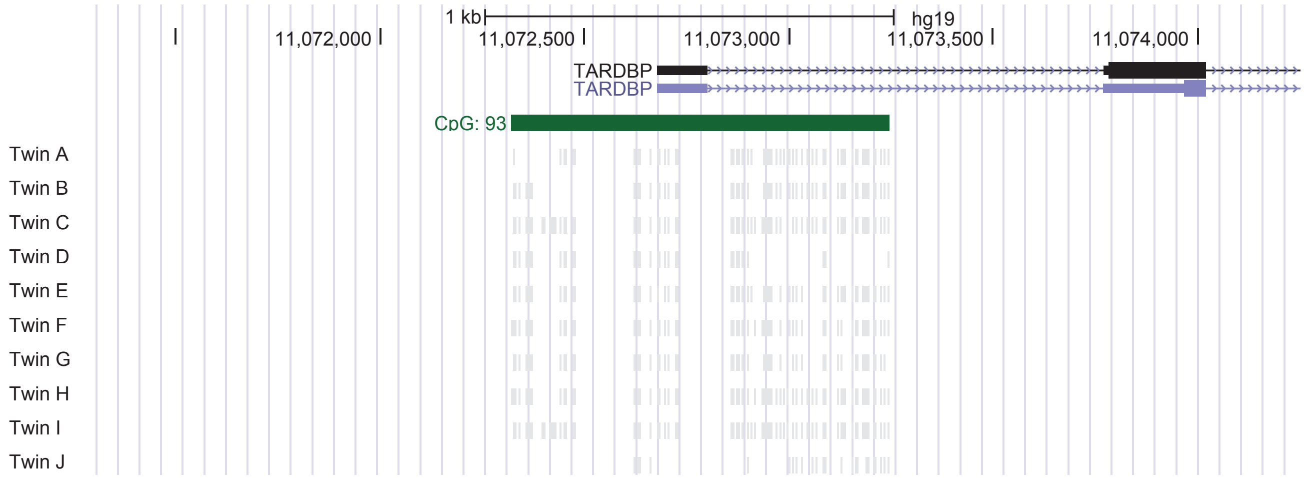

UBQLN2 - Chr X

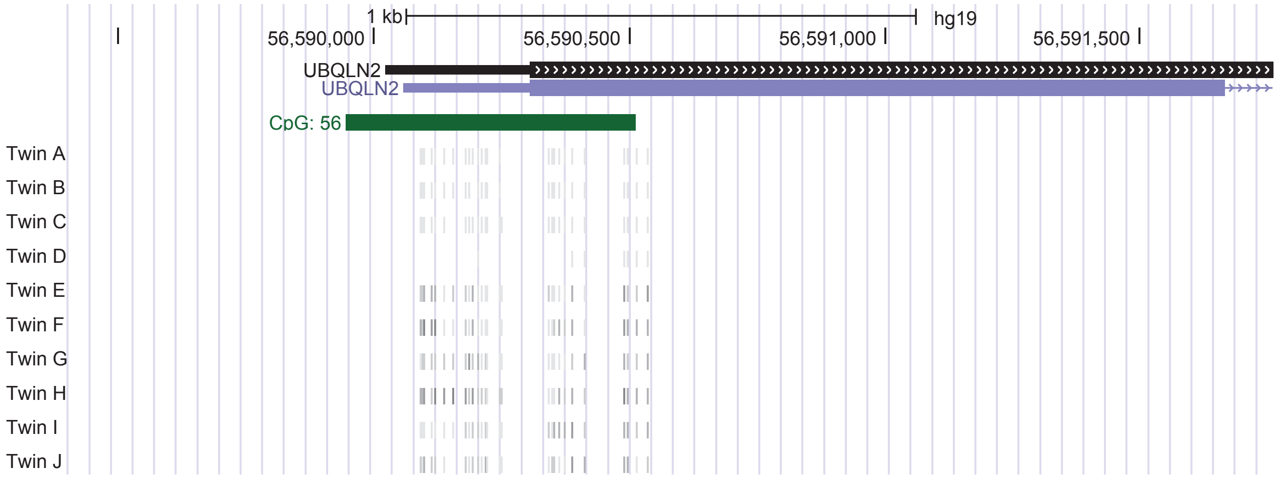

VAPB -Chr 20

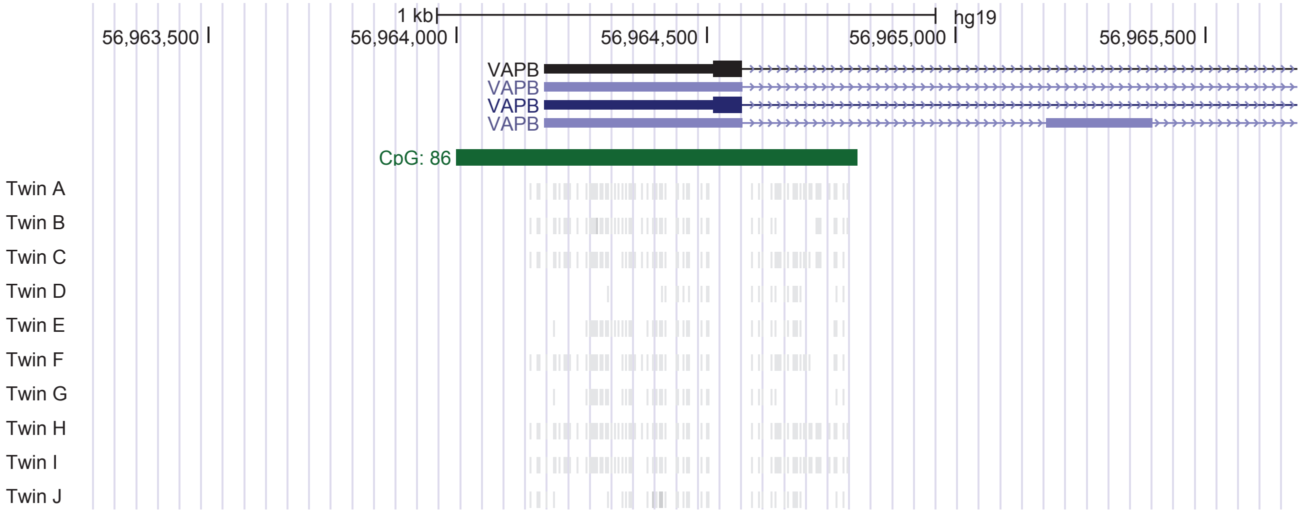

VCP - Chr 9

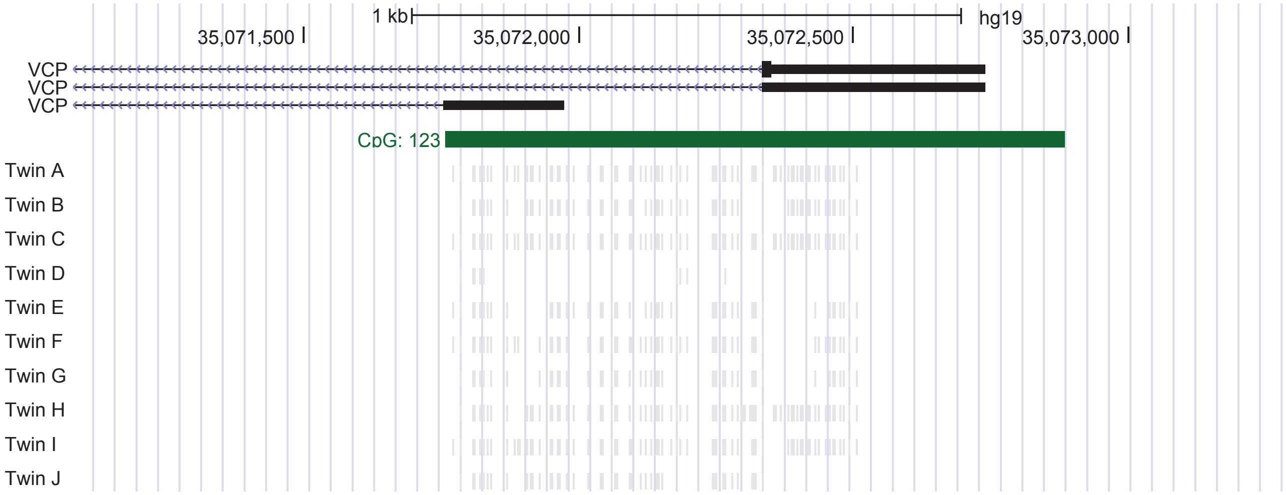

Gene	Twin	Average %5mc across region	Number of Sites >=10X	Average coverage >= 10x
ADAR	A	0.05	51	26
	B	1.58	51	16
	C	3.33	51	49
	D	0.6	43	22
	E	1.09	43	15
	F	0	35	21
	G	1.2	42	14
	H	0.15	51	25
	I	3.88	27	20
	J	0.88	50	24
ALS2	A	1.85	54	26
	B	3.44	47	17
	C	9.35	59	31
	D	4.23	29	16
	E	3.37	30	15
	F	2.33	43	15
	G	6.84	19	12
	H	9.75	55	17
	I	4.67	25	12
	J	3.15	51	17
ATXN2	A	0.71	210	20
	B	1.45	155	14
	C	3.62	247	30
	D	4.92	91	13
	E	2.22	109	13
	F	1.6	116	16
	G	2.79	79	14
	H	4.17	171	19
	I	7.46	63	14
	J	3.42	194	18
C9orf72	A	0.07	74	33
	B	1.02	70	21
	C	1.07	78	44
	D	0.84	66	24
	E	0.12	57	19
	F	0.15	76	19
	G	0.86	59	15
	H	1.62	80	31
	I	0.47	55	15
	J	0.27	84	21
FUS	A	0.54	71	25
	B	1.19	43	18
	C	0.76	67	40
	D	0.68	57	21
	E	0.84	64	14
	F	0.66	46	23
	G	1.82	29	17
	H	0.39	60	26
	I	2.11	38	13
	J	0.65	59	18
OPTN	A	2.82	76	25
	B	3.49	68	16
	C	3.44	89	38
	D	6.3	63	17
	E	8.38	37	16
	F	6.69	50	20
	G	4.87	25	15
	H	4.77	72	21
	I	1.5	29	17
	J	5.23	80	20
PFN1	A	0.15	250	28
	B	1.24	200	19
	C	0.88	250	40
	D	1.16	163	24
	E	0.87	168	17
	F	0.39	206	22
	G	1.01	115	17
	H	0.38	230	27
	I	1.03	131	15
	J	0.88	191	22

Gene	Twin	Average %5mc across region	Number of Sites >=10X	Average coverage >= 10x
SETX	A	0.62	65	23
	B	0.11	38	19
	C	1.24	72	36
	D	0.43	46	16
	E	0	28	15
	F	0.14	65	14
	G	1.02	41	14
	H	0.32	67	21
	I	1.54	28	15
	J	0.17	58	16
SOD1	A	2.48	74	27
	B	5.38	56	18
	C	3.27	87	42
	D	2.54	61	28
	E	3.1	52	19
	F	2.09	84	21
	G	4.34	51	16
	H	2.51	79	31
	I	3.69	44	12
	J	2.37	87	22
SPG11	A	0.18	62	27
	B	1.31	44	20
	C	0.88	62	38
	D	0.96	49	16
	E	1.04	34	16
	F	4.25	36	15
	G	0	25	12
	H	0.48	62	20
	I	0.63	29	12
	J	0.66	57	21
TARDBP	A	0.05	83	27
	B	1.27	80	19
	C	1.44	103	32
	D	1.09	64	23
	E	0.58	61	17
	F	0.12	70	21
	G	0.8	40	14
	H	0.33	94	25
	I	0.99	14	19
	J	0.8	96	20
UBQLN2	A	0.26	38	23
	B	1.29	26	15
	C	1.29	35	27
	D	0.34	20	20
	E	20.65	39	22
	F	21.78	33	26
	G	20.44	34	20
	H	32.33	39	35
	I	21.03	35	20
	J	18.85	34	28
VAPB	A	0.2	103	25
	B	1.19	71	15
	C	1.42	103	32
	D	0.91	55	20
	E	1.49	47	19
	F	0.2	82	18
	G	1.21	26	17
	H	1.59	103	23
	I	0	31	13
	J	0.62	100	18
VCP	A	0.08	108	23
	B	1.07	71	17
	C	1.32	123	35
	D	0	42	13
	E	0.6	46	15
	F	0	72	13
	G	1.71	35	12
	H	0.56	100	15
	I	1.28	44	14
	J	0.61	89	17
