# Supplementary material for: Epigenetic differences between monozygotic twins discordant for amyotrophic lateral sclerosis (ALS) provide clues to disease pathogenesis
Source: PLoS One. 2017 Aug 10;12(8):e0182638. doi: 10.1371/journal.pone.0182638 (PMC5552194; doi:10.1371/journal.pone.0182638)
Supplement: S3 File — (PDF) [file pone.0182638.s006.pdf]

Supplementary File 2: Guide to searching the twin-related methylation status of a gene of interest (in Supplementary Tables 2 and 3)

These tables provide information on CpG sites that are commonly differentially methylated in all ALS cases vs all matched unaffected twin siblings (Table S2), or are differentially methylated between twins in one or more pair (Table S3).

Table S2: CpG sites that are differentially methylated in all ALS patients vs all matched unaffected twin siblings.

To find whether your gene of interest is in this list, search (Edit/Find) the Excel spreadsheet "All ALS vs nonALS" in Table S2 using the official gene symbol in uppercase text. The gene name can appear multiple times if the gene has a number of methylation differences. To find all instances of this gene name, apply Data/Filter to the entire sheet, then on the dropdown menu in the "Gene name" title, type the gene name in the Search box.

For example, to ask whether the gaba receptor subunit d is present, search for "GABRD". If the gene you are looking for is not found in this sheet, it is most likely that it was did not differ in methylation between cases and controls. It is also possible that your gene was not captured by RRBS. All raw data has been made available for you assess this.

If the gene is present, the spreadsheet will take you to the row location of the gene. In the same row you will find information on:

1. The genomic location (the chromosome number. and the genomic coordinate of the CpG). hg19 = the 19th version of the Human Reference Genome

2. The significance values of the difference (*q* value (FDR corrected p value, with values less than 0.01 considered significant)

3. The % difference in methylation between affected and unaffected individuals. A positive value means the site is hypermethylated in the affected (ALS) relatives compared to the unaffected (nonALS) individuals, and a negative value means the site is hypomethylated in affected relatives compared to unaffected.

4. The genomic annotation (i.e., whether the site is situated in an intron, exon, etc or between genes (intergenic).

5. The Genbank accession number of the affected gene.

Searching for GABRD in Table S2 should produce this result:

Gene	Genomic location	*q* value	Methylation difference % (cases - controls)	Genomic annotation	Genbank accession no.
GABRD	chr1.1956194	2.01E-05	-22.88	intron	NM_000815

This indicates that the GABRD methylation at this site is situated in an intron, is significantly different between groups, and is less methylated in the ALS group than the non-ALS group.

Table S3: CpG sites that are differentially methylated between twins in one or more twin pair.

Table S3 contains information on CpG sites that we have defined as methylation outliers in each twin pair, i.e., there is a large difference in methylation between genetically identical affected (ALS) and unaffected (nonALS) co-twins.

Each twin pair has its own list of outliers. To find whether your gene of interest is in any of these lists, search the "All_pairs" sheet in Table S3 using the official gene symbol in uppercase text.

For example, to ask whether the glutamate receptor subunit zeta-1 is present, search for "GRIN1". If your gene name is not on the list, this likely means it was not identified as an outlier in this study (i.e., no CpGs in your gene of interest were different between ALS affected twins and their unaffected co-twin). Again though, it is possible that your gene was not captured by RRBS and so you will need to access the raw data to determine this.

If the gene is present, the spreadsheet will take you to the rows including that gene, and show in which twin pair(s) it was identified as an outlier.

Searching for GRIN1 in Table S2 should produce this result:

**Gene name**	**Location (hg19)**	**Table S2A - Pair 1**	**Table S2B - Pair 2**	**Table S2C - Pair 3**	**Table S2D - Pair 4**	**Table S2E - Pair 5**	**# Pairs in common**	**Genomic annotation**	**Genbank accession no.**
GRIN1	chr9.140034340		1				1	intron	NM_007327
GRIN1	chr9.140044074				1		1	intron	NM_007327
GRIN1	chr9.140053706		1				1	intron	NM_007327
GRIN1	chr9.140056997		1				1	intron	NM_007327
GRIN1	chr9.140057165	1					1	exon	NM_007327
GRIN1	chr9.140057894			1			1	intron	NM_007327
GRIN1	chr9.140057908			1			1	intron	NM_007327
GRIN1	chr9.140062520					1	1	3'UTR	NM_007327

If the outlier is present in a particular twin pair comparison it will be denoted as a '1' in the column pertaining to a particular twin pair. The column '# Pairs in common' shows how many twin pairs in whom that site was affected.

The example above shows that there are multiple outliers identified at GRIN1, in this case in all twin pairs, but no single CpG site is in common (so the # Pairs are all N=1 here).

To view the specifics of individual twin pair differences at any gene listed in the all-pairs sheet (i.e., the percentage methylation at the particular site), search the gene again (as per the instructions for Table S2) on the tab for the relevant twin pair. For example, for the GRIN1 finding for twin Pair 5 (sheet tab "TableS2E – Pair 5") is shown below. The ALS twin has 21.2% methylation at this 3'UTR site, while the nonALS twin has 64.3% methylation.

**Gene name**	**Location (hg19)**	**% methylation TwinI (ALS)**	**% methylation TwinJ (nonALS)**	**% difference**	**Genomic annotation**	**Genbank accession no.**
GRIN1	chr9.140062520	21.2	64.3	-43.1	3'UTR	NM_007327
