# Supplementary material for: Epigenetic differences between monozygotic twins discordant for amyotrophic lateral sclerosis (ALS) provide clues to disease pathogenesis
Source: PLoS One. 2017 Aug 10;12(8):e0182638. doi: 10.1371/journal.pone.0182638 (PMC5552194; doi:10.1371/journal.pone.0182638)
Supplement: S1 Table — (PDF) [file pone.0182638.s007.pdf]

Table S1: CpG coverage in RRBS libraries

	CpG sites covered	Average CpG coverage	CpG sites covered at ≥10x	CpG sites covered at ≥20x
Twin A	3,531,499	21	2,172,290	1,459,603
Twin B	3,254,600	16	2,063,986	1,117,291
Twin C	3,875,237	39	2,627,119	2,349,715
Twin D	3,478,970	20	2,254,292	1,334,981
Twin E	3,212,696	16	2,093,009	1,004,669
Twin F	3,031,226	22	2,315,834	1,442,499
Twin G	2,999,977	13	1,817,835	677,885
Twin H	3,213,850	28	2,467,025	1,847,004
Twin I	3,637,416	12	1,789,062	738,341
Twin J	3,241,506	23	2,411,208	1,713,635
